# Interleukin-6-dependent growth in a newly established plasmablastic lymphoma cell line and its therapeutic targets

**DOI:** 10.1038/s41598-017-10684-5

**Published:** 2017-08-31

**Authors:** Sohtaro Mine, Tsunekazu Hishima, Akihiko Suganuma, Hitomi Fukumoto, Yuko Sato, Michiyo Kataoka, Tsuyoshi Sekizuka, Makoto Kuroda, Tadaki Suzuki, Hideki Hasegawa, Masashi Fukayama, Harutaka Katano

**Affiliations:** 1 Department of Pathology, National Institute of Infectious Diseases, Tokyo, Japan; 2 Department of Pathology, Graduate School of Medicine, The University of Tokyo, Tokyo, Japan; 3 Department of Pathology, Tokyo Metropolitan Komagome Hospital, Tokyo, Japan; 4 Department of Infectious Diseases, Tokyo Metropolitan Komagome Hospital, Tokyo, Japan; 5 Pathogen Genomic Center, National Institute of Infectious Diseases, Tokyo, Japan

## Abstract

Plasmablastic lymphoma (PBL) is a rare, highly aggressive subtype of non-Hodgkin lymphoma with plasma-cell differentiation occurring typically in immune-suppressed patients such as those with AIDS. This study reports the establishment and characterization of a new cell line, PBL-1, derived from a patient with AIDS-associated PBL. Morphological assessment of PBL-1 indicated plasma-cell differentiation with a CD20(−) CD38(+) CD138(+) immunophenotype and IgH/c-myc translocation. The cell line harbours Epstein-Barr virus, but a 52.7-kbp length defect was identified in its genome, resulting in no expression of viral microRNAs encoded in the BamHI-A Rightward Transcript region. Importantly, supplementation of culture medium with >5 ng/mL of interleukin-6 (IL-6) was required for PBL-1 growth. Starvation of IL-6 or addition of tocilizumab, an inhibitory antibody for the IL-6 receptor, induced apoptosis of PBL-1. Transduction of IL-6 into PBL-1 by lentivirus vector induced autologous growth without IL-6 supplementation of culture medium. These data indicate the IL-6 dependency of PBL-1 for proliferation and survival. mTOR inhibitors induced cell death effectively, suggesting mTOR in the IL-6 signalling pathway is a potential therapeutic target for PBL. This established PBL cell line will be a useful tool to further understand the pathophysiology of PBL and aid the future development of PBL treatment.

## Introduction

Plasmablastic lymphoma (PBL) is a rare distinct clinicopathological entity of large B-cell malignant lymphoma, occurring typically in HIV-1 infected or other immunodeficient patients and is generally associated with Epstein–Barr virus (EBV) infection^1, 2^. The incidence of PBL has been estimated as 2–8% of all cases of AIDS-related lymphomas^3, 4^. Histologically, PBL cells have a large, round-to-irregular nucleus with a prominent nucleoli and perinuclear halo, as well as a variable amount of cytoplasm that is deeply basophilic^2^. PBL cells do not express an immunophenotype typical for B-cell (surface and cytoplasmic immunoglobulin, CD19, CD20, CD79a) or T-cell (CD3, CD4, CD8) markers. Instead, several markers of lymphocyte activation (CD30, CD38, CD70, human leukocyte antigen DR) and plasma-cell differentiation (CD138, Blimp-1) are usually detected.

EBV has been detected in 66–74% of PBL cases^5, 6^, suggesting it might play an important role in the tumorigenesis of PBL. EBV is a member of the oncogenic human gamma-herpesvirus family, and has been detected in Hodgkin lymphoma, Burkitt lymphoma, nasopharyngeal carcinoma, post-transplantation lymphoproliferative disorder, and gastric cancer. Approximately 40% of AIDS-related lymphoma cases are positive for EBV^7^. In PBL cases, EBV has been detected as Latency I, expressing EBV-encoded small RNAs (EBERs) and EBV-encoded nuclear antigen 1 (EBNA-1). Although integration of EBV in the host chromosome was observed in several cell lines established from EBV-associated lymphoma with persistent infection^8–13^, there has been no report of an EBV integrated case of PBL.

The prognosis of PBL is very poor, and a standard therapy for PBL has not been established^1, 14^. Currently, a combination of antiretroviral therapy^15, 16^ with a CHOP (cyclophosphamide, doxorubicin, vincristine, and prednisolone)-like regimen or more aggressive regimen including CODOX-M/IVAC (cyclophosphamide, vincristine, doxorubicin, high-dose methotrexate alternating with ifosfamide, etoposide, and high-dose cytarabine), dose-adjusted EPOCH (infusional etoposide, vincristine and doxorubicin with bolus cyclophosphamide and prednisone), or HyperCVAD (hyper-fractionated cyclophosphamide, vincristine, doxorubicin, and dexamethasone alternating with methotrexate and cytarabine) are considered first-line therapies^1, 17, 18^. Recently, cases treated with chemotherapy in combination with bortezomib, a proteasome inhibitor, have been reported^19–24^.

Immortalized cell lines are useful tools to investigate disease pathogenesis and to evaluate the effects of therapeutic agents. To the best of our knowledge, there has been no report describing a well-characterized PBL cell line established from typical PBL cases. In this study, we established a PBL cell line from a case of AIDS-associated PBL, and demonstrated its interleukin 6 (IL-6)-dependent growth. In addition, the treatment effect of therapeutic agents was investigated.

## Results

### Establishment of a PBL cell line

At the beginning of the primary culture of tumour cells from the patient ascites, cells grew in RPMI 1640 culture medium supplemented with 10% patient ascites, 10% foetal bovine serum (FBS), 10 ng/mL insulin, and 10 ng/mL of transferrin at 37 °C with 5% CO_2_. After several passages, autologous growth was observed. However, when the ascites was removed from the culture medium, the cells did not grow and became apoptotic. Therefore, we speculated that certain growth factor(s) were required for cell growth. Cytokines in the patient’s ascites and the supplemented cultured medium were analysed by Luminex. A high amount of IL-6 was detected in the ascites (Supplementary Figure 1). Supplementation of RPMI 1640 containing 10% FBS with 5 ng/mL of IL-6 induced cell proliferation, and the percentage of live cells increased to >90%. Single cell cloning was performed successfully in medium containing IL-6 using a limiting dilution method. Five single clones with similar morphological phenotypes were obtained, and one of single cell-cloned cells was designated as PBL-1 and analysed.

### Morphological, immunological, and genetic character of PBL-1

**Figure 1:**
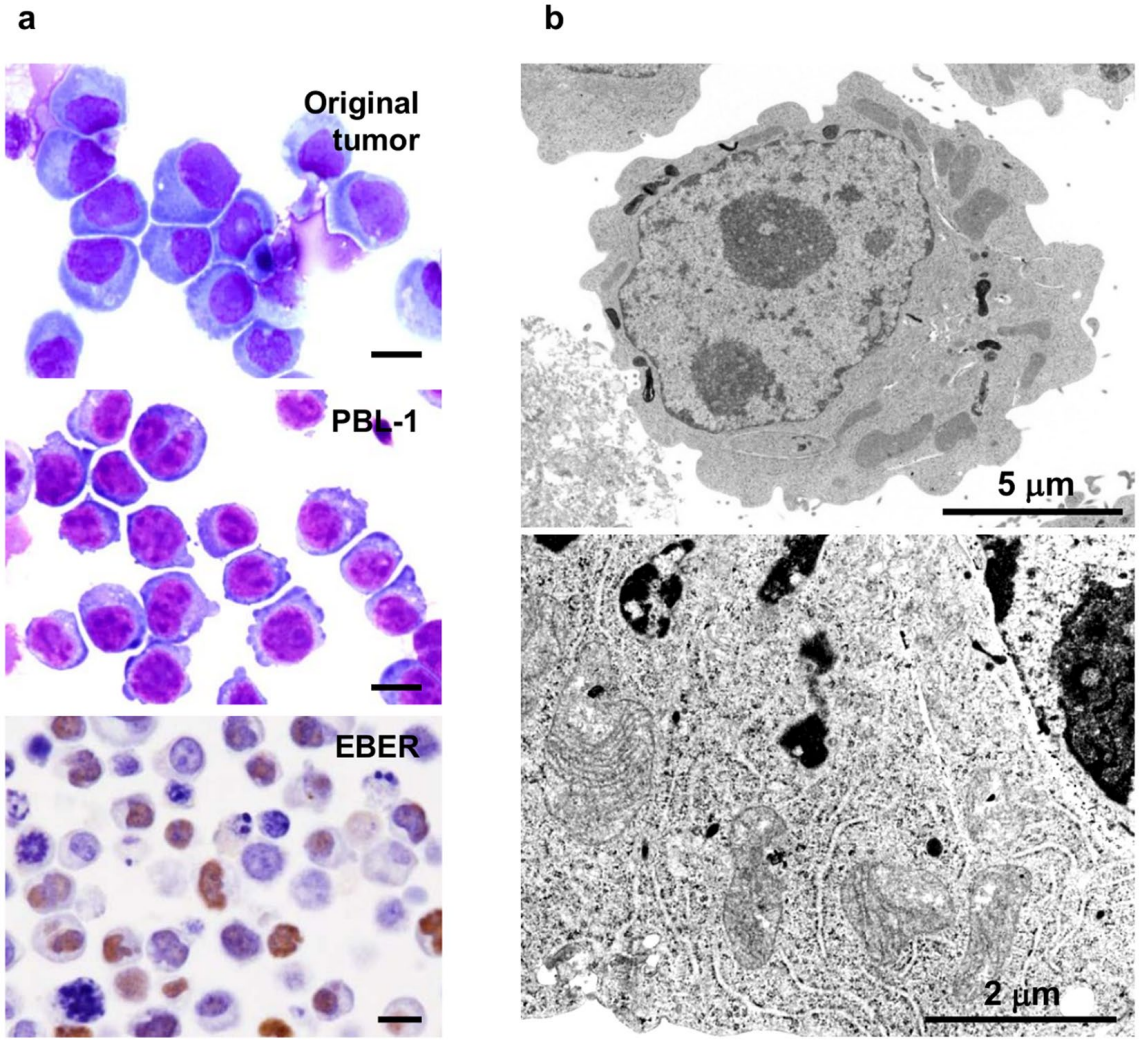
Morphology of PBL-1. (**a**) Microscopic view of PBL-1. Giemsa staining of original tumour cells in the ascites of an HIV positive PBL patient (upper panel) and PBL-1 (middle panel), and EBER-1 *in situ* hybridization on a formalin-fixed paraffin-embedded sample of a PBL-1 cell pellet (lower panel) are shown. Each black bar indicates 10 μm. EBV-positive lymphoma tissues from other unrelated patients also showed positive signals in EBER-1 *in situ* hybridization (data not shown). (**b**) Transmission electron microscopy of PBL-1. Chromatin concentrated in the centre and margin of the nucleus, with a wheel-like appearance (upper panel). Abundant rough endoplasmic reticulum is observed in the perinuclear region (lower panel).

Giemsa staining revealed that PBL-1 had basophilic cytoplasm with a perinuclear halo and an unevenly distributed and enlarged nucleus with marked nucleoli (Fig. 1a). Many large cells had two nuclei. Electron microscopy demonstrated abundant rough endoplasmic reticulum around the nucleus (Fig. 1b). Flow cytometry and immunofluorescence analysis revealed that PBL-1 were positive for CD38, CD138, CD45RA, Blimp-1, MDM2, MyD88, gp80 (IL-6Ra), and gp130 (IL-6 signal transducer), and were negative for CD3, CD4, CD8, CD10, CD30, CD19, CD20, and Ig light chains λ and κ (Supplementary Figure 2). This immunophenotype suggests that the origin of PBL-1 was a plasmablast. *In situ* hybridization demonstrated that PBL-1 were positive for EBER-1 (Fig. 1a). PCR analysis indicated that PBL-1 were positive for EBV, and negative for Kaposi’s sarcoma-associated herpesvirus (KSHV) and HIV-1 (Supplementary Figure 3a). The multivirus real-time RT-PCR system showed that PBL-1 cells were positive for only EBV and negative for more than 160 other viruses including HIV-1 and KSHV (Supplementary Table 1).

Chromosomal analysis revealed a complex karyotype — the majority of the cells were diploid with modal 82 chromosomes (Supplementary Figure 4). Southern blotting analysis indicated an IgH gene rearrangement, and BioMed2 analysis revealed an immunoglobulin kappa gene rearrangement, indicating a B cell linage (Supplementary Figure 4). Fluorescence *in situ* hybridization (FISH) with a triple colour-dual fusion probe of IgH and c-myc revealed that PBL-1 had an IgH/c-myc rearrangement (Supplementary Figure 4d), whereas there were no bcl-2 or bcl-6 gene splits (data not shown). PCR and sequence analysis identified a break point in the IgH/c-myc rearrangement that was upstream of exon 1 of c-myc and downstream of the IgH regulatory region (Supplementary Figure 4e and f). Immunofixation electrophoresis of supernatants from the PBL-1 culture medium revealed that PBL-1 secreted no immunoglobulin heavy or light chains (data not shown). Sequence analysis of c-myc, p53, and myd88 revealed these genes had no recurring point mutations in PBL-1 compared with reference sequences (data not shown).

### EBV infection in PBL-1

**Figure 2:**
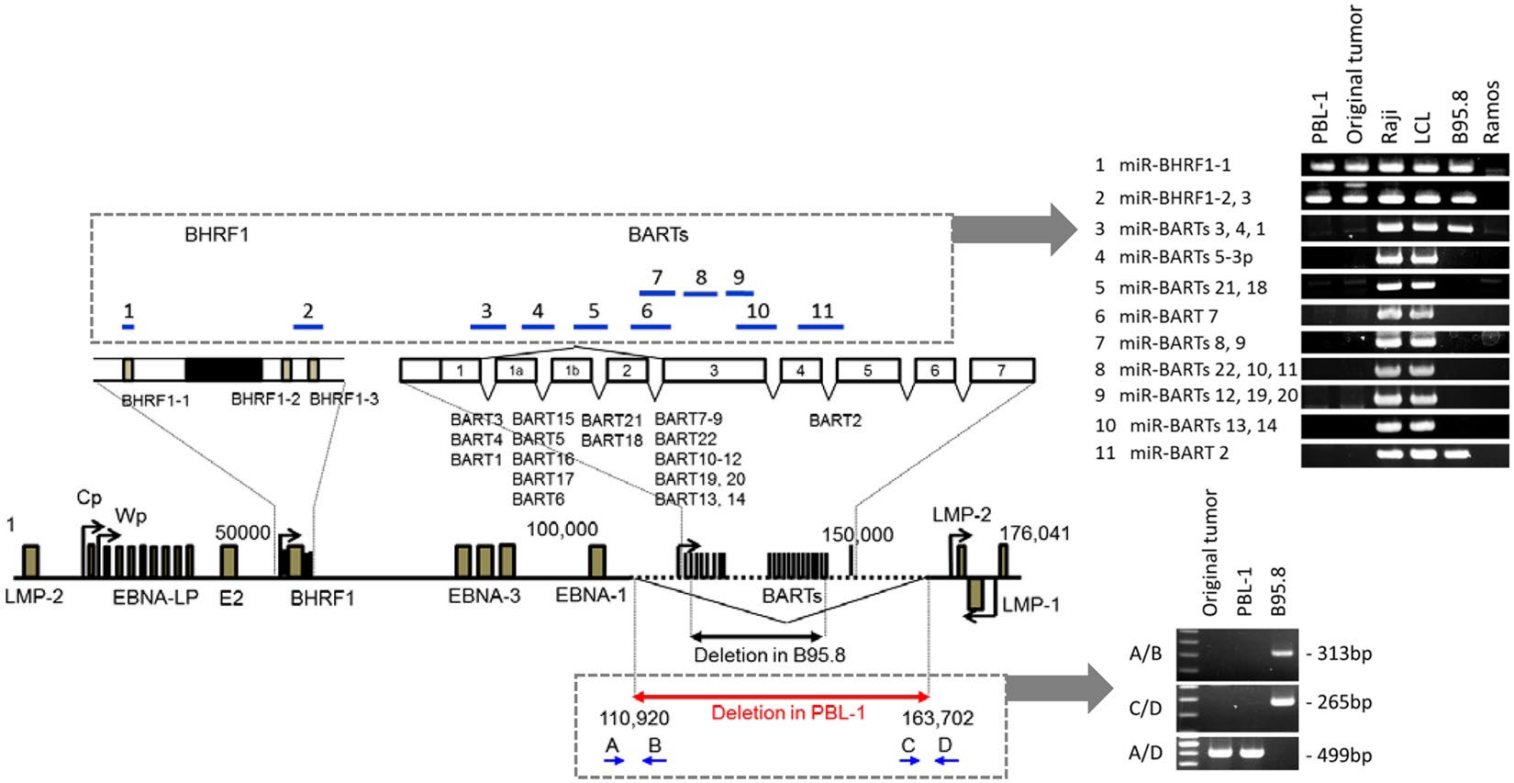
Deletion of EBV genome in PBL-1. Genetic map of EBV is shown (left). Deleted region of PBL-1 is indicated by the red arrow. Deleted region in B95.8 EBV is indicated by the lower black arrow. The right panels show results of PCR analysis for EBV DNA encoding BHRF1 and BART miRNA clusters (upper panel), and PCR of the deletion site of EBV in PBL-1 (lower panel). Upper blue lines in the EBV genetic map indicate PCR amplicons for EBV DNA encoding miRNAs in the right upper panel. Lower blue arrows in EBV genetic map are PCR primers used for PCR analysis of the breakpoint (right lower panel).

**Figure 3:**
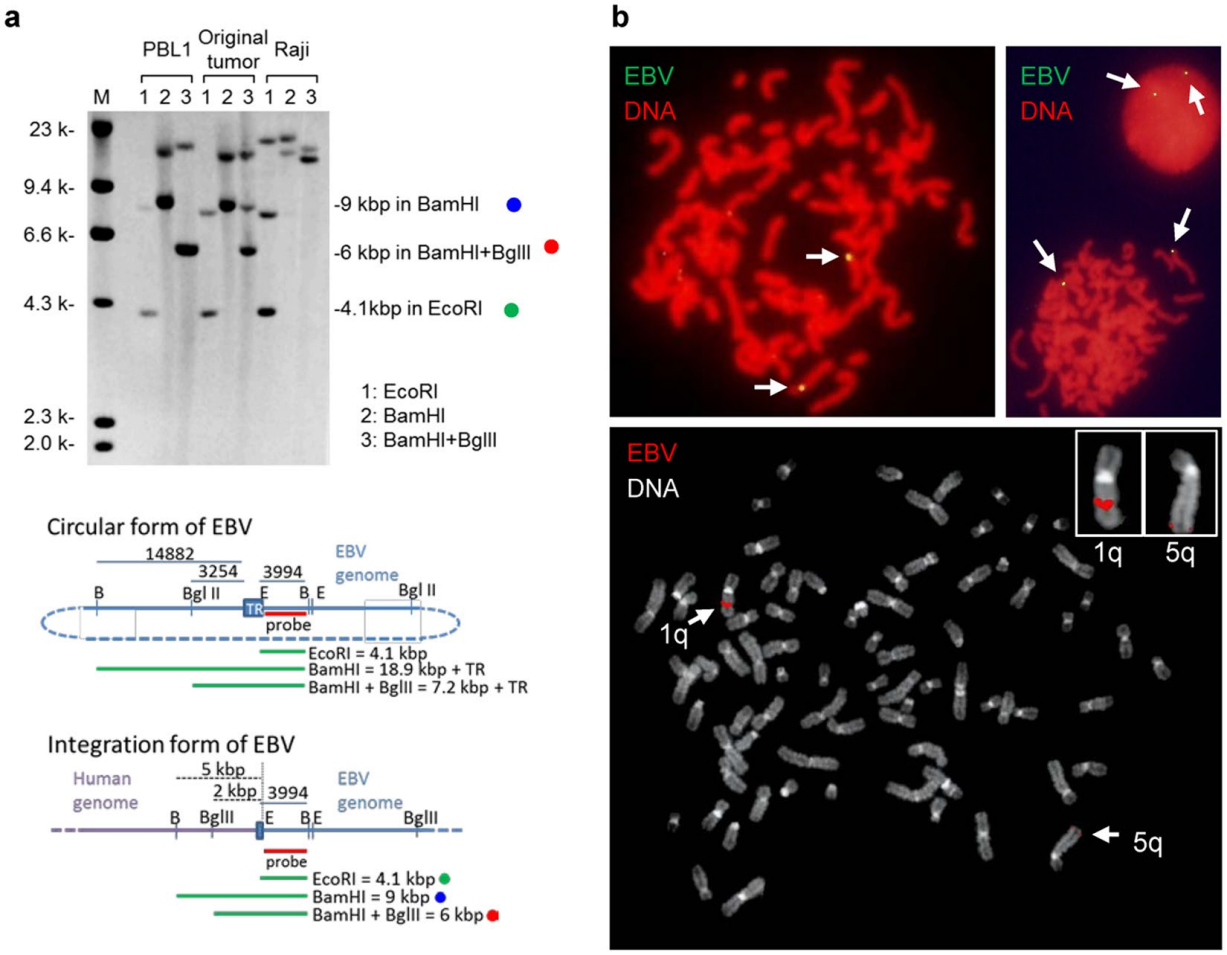
EBV genome in PBL-1. (**a**) Southern blot analysis using DIG-labelled EBV-Bam-L probe. 1: *Eco*RI, 2: *Bam*HI, and 3: *Bam*HI+ *Bgl*II -digested DNA. Actual observed sizes are 4.1, 9.0, and 6.0 kbp in lanes 1, 2, and 3, respectively. In the lower two panels of maps of TR in EBV, the predicted size of bands in Southern blot hybridizations are shown if the circular (upper) or integration (lower) form of EBV is predominant in PBL-1. Green, blue, and red dots correspond to those in the top panel. (**b**) FISH for EBV in chromosomes in the metaphase and interphase of PBL-1. EBV genome was labelled by green (Alexa 488, upper panels) or red fluorescence (Rhodamine, lower panel). Chromosomal DNA was counterstained with propidium iodide (red, upper panels) or 4′,6-diamidino-2-phenylindole (white, lower panel). Two or four yellow or red small dots were observed per cell (arrows, upper panels). Chromosomal analysis revealed that EBV signals were found in 1q and 5q (arrows, lower panel and insets).

RT-PCR revealed that PBL-1 was positive for EBER-1 and EBNA-1 mRNA but negative for EBNA-2, latent membrane protein 1 (LMP-1), LMP-2A, and LMP-2B mRNA, indicating that the EBV status in PBL-1 was Latency I (Supplementary Figure 3c). RT-PCR also revealed Qp promotor usage and that the Cp and Wp promoters were not active in PBL-1 (Supplementary Figure 3c). EBV genotype-specific PCR analysis revealed that EBV in PBL-1 was genotype 1 (Supplementary Figure 3b). Reactivation of EBV was investigated by the addition of 12-O-tetradecanoylphorbol-13-acetate (TPA) or Ig in the culture supernatant of PBL-1. Real-time PCR analysis showed that the EBV copy number was stable at 1.26 copies/cell in PBL-1 regardless of stimulation by TPA or Ig. To investigate the expression of EBV-encoded miRNA in PBL-1, next generation sequencing (NGS) was performed on small RNA extracted from PBL-1. Surprisingly, NGS detected only 2 reads of miR-BHRF1, but no reads of miR-BART in 2,112,810 reads, while human miRNA were detected abundantly (Supplementary Table 2). NGS also detected only 1 read of miR-BHRF1 in 1,426,476 reads from an RNA sample of the original tumour. PCR with primers designed for the region of miR-BART clusters in the EBV genome demonstrated a defect in the region (Fig. 2). Genome walking and PCR analysis revealed a deletion from 110,920 to 163,702 in the EBV genome of PBL-1 (numbering is based on strain M81, GenBank accession no. KF373730), indicating a complete deletion of miR-BART clusters (Fig. 2). Southern blot analysis with the 5′ site of the EBV genome detected a band of unexpected size for the circular form of EBV (Fig. 3a). FISH using an EBV-specific probe detected 2–4 dots of positive signals per cell in chromosomes 1q and 5q in the metaphase or interface (Fig. 3b). In addition, digital PCR analysis showed that EBV copy number in PBL-1 was stable at 1.67–2.7 copies per 1 beta-actin genome regardless of chemical or immunological stimulation (Supplementary Figure 3d and e). These data suggest an integrated form of the EBV genome in PBL-1.

### PBL-1 is dependent on IL-6 for proliferation and survival

**Figure 4:**
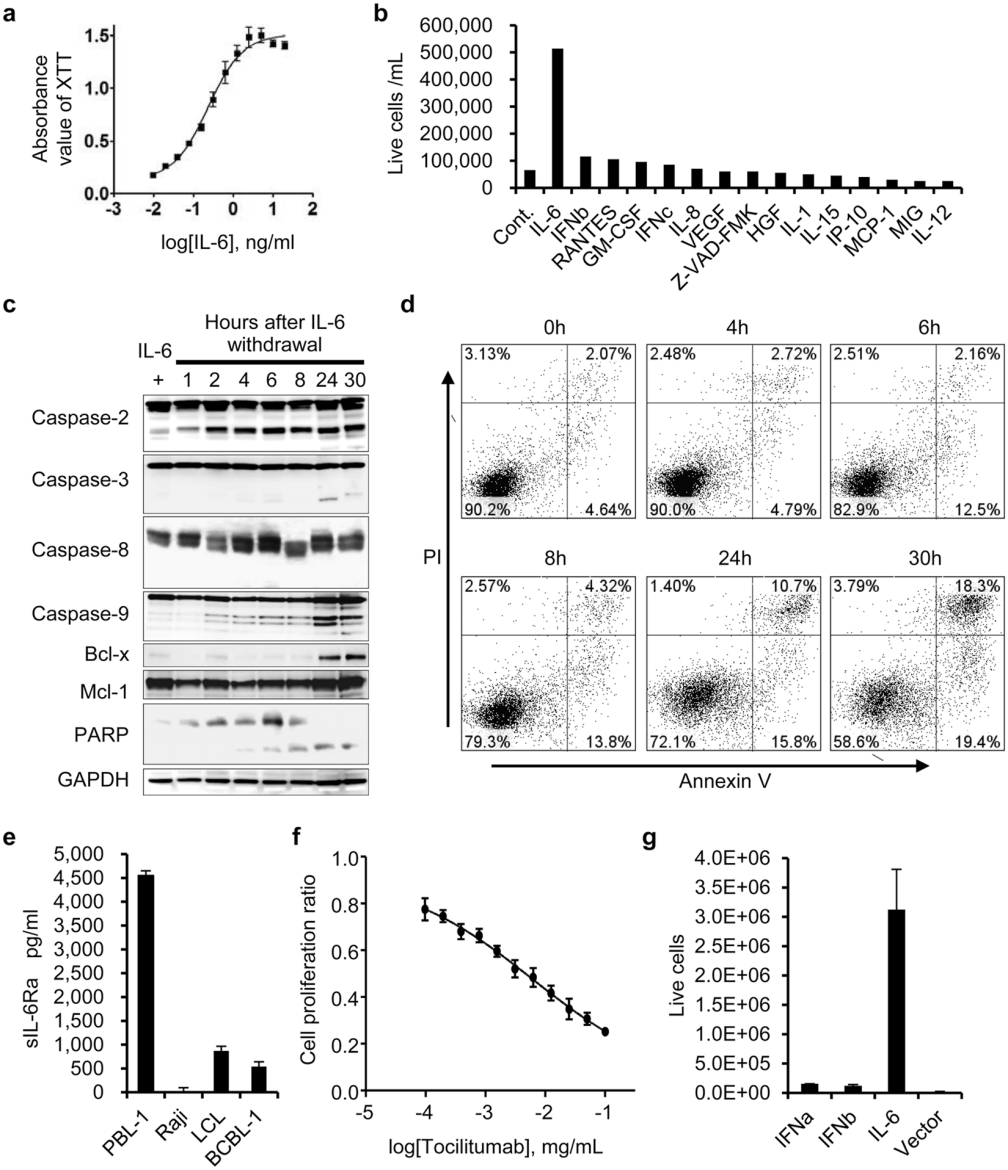
IL-6-dependent growth of PBL-1. (**a**) IL-6 concentration and cell viability by XTT assay. Absorbance of XTT assay correlated with the concentration of IL-6. (**b**) Comparison with other cytokines. PBL-1 proliferated in medium supplemented with IL-6, but not other cytokines. (**c**) Western blot analysis of apoptosis-associated proteins. (**d**) Flow cytometry for Annexin V and propidium iodide (PI) apoptosis assay in PBL-1 cultured in medium without IL-6. Hours after IL-6 withdrawal are shown at the top of each panel. (**e**) sIL-6R levels in culture supernatants. High levels of sIL-6Ra were detected in the supernatant of PBL-1 cells. (**f**) XTT assay of PBL-1 treated with tocilizumab, an anti-IL-6R monoclonal antibody. Tocilizumab decreased the cell proliferation ratio of PBL-1 in a dose dependent manner. (**g**) Proliferation of PBL-1 transduced with lentivirus expressing IL-6. Transduction of IL-6 by lentivirus vector, but not vector alone, or IFN-α and IFN-β expressing lentiviruses, induced the autologous proliferation of PBL-1. The error bars in each panel represent the standard deviation of the measurements.

PBL-1 showed constant growth in medium supplemented with 5 ng/mL of IL-6. The XTT assay showed the IL-6-dose-dependent viability of PBL-1 (EC_50_ = 0.237 ng/mL) (Fig. 4a). The XTT assay and cell viability counting indicated that other cytokines including IL-8, IL-4, IL-11, Monocyte Chemotactic Protein-1 (MCP-1), and oncostatin M had no effect on PBL-1 viability (Fig. 4b). Western blot analysis showed that starvation of IL-6 led to PBL-1 apoptosis and the cleavage of caspases-2, -3, -9, and PARP (Fig. 4c). Flow cytometry revealed that annexin V expression was induced at 6 hours after IL-6 starvation in PBL-1 (Fig. 4d), and BrdU assay showed a decrease of DNA synthesis in PBL-1 at 24 hours (Supplementary Figure 5a). IL-6 binds to soluble or membranous IL-6 receptor (or gp80, IL-6Ra), and this IL-6/IL-6Ra complex binds to two molecules of gp130, a ubiquitously expressed membranous protein^25^. The IL-6/IL-6R/gp130 complex induces activation of the Jak/Signal Transducers and Activator of Transcription (STAT) 3 pathway^26–28^. Flow cytometry analysis revealed the expression of gp80 and gp130 in PBL-1 (Supplementary Figure 2). In addition, enzyme-linked immunosorbent assay demonstrated that PBL-1 secreted a high concentration of soluble IL-6Ra (4,482 pg/mL in the supernatant of PBL-1, Fig. 4e). Tocilizumab, an anti-IL-6 receptor antibody, inhibited mitochondrial metabolic activity and DNA synthesis in PBL-1 in a dose-dependent manner (IC_50_ = 6.11 μg/mL, Fig. 4f, and Supplementary Figure 5b), and induced apoptosis (Supplementary Figure 5c). Lentiviral transduction of PBL-1 with human IL-6, but not a control-gene or vector-transduced cells, without supplementation of IL-6 induced significant autologous proliferation and survival of the transduced cells (p < 0.01) (Fig. 4g). These data demonstrated a dependency on IL-6 for PBL-1 growth.

### IL-6/Jak/STAT3 and PI-3K/Akt/mTOR pathways in PBL-1

**Figure 5:**
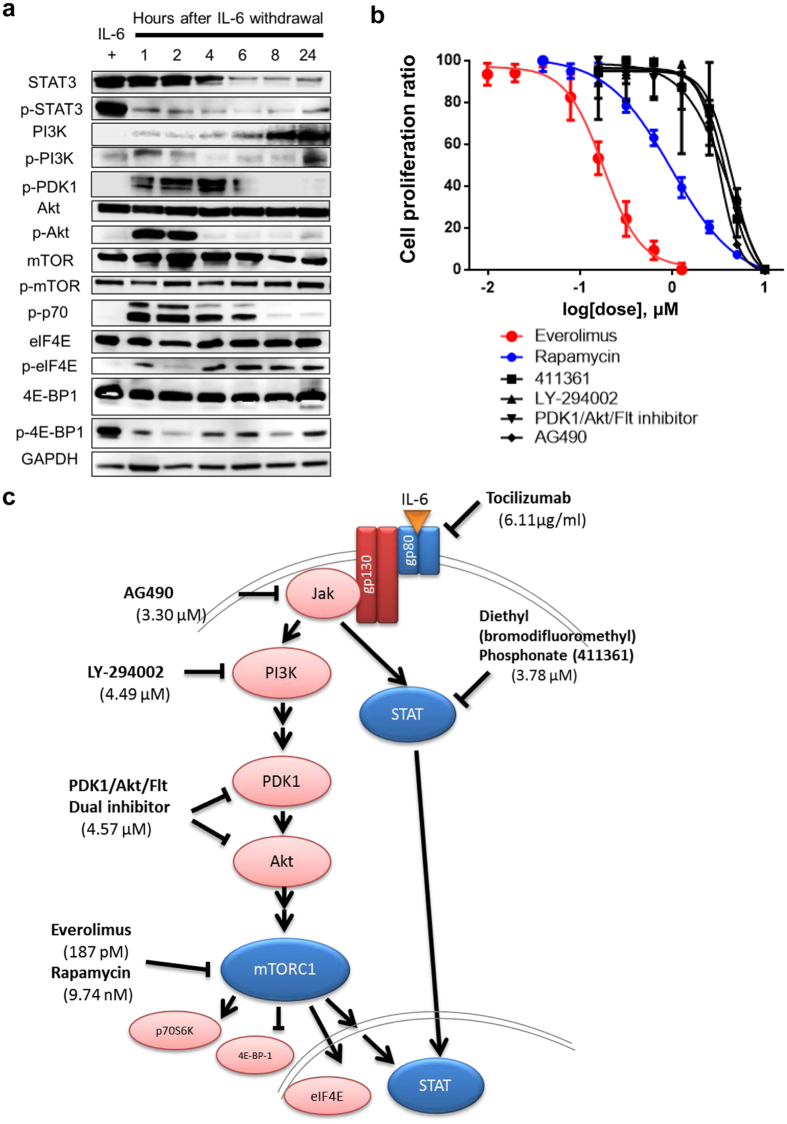
IL-6 signal pathway in PBL-1. (**a**) Western blot analysis of IL-6 signal-associated proteins. (**b**) Effect of inhibitors for Jak/STAT and PI3K/Akt/mTOR pathways on PBL-1 by XTT assay. Cell proliferation ratio (%) in XTT assay and the concentration of each drug are indicated on the vertical and horizontal axes. All inhibitors were added to medium supplemented with 5 ng/mL IL-6. The error bars represent the standard deviation of the measurements. (**c**) The IL-6 signal pathway in PBL-1. Inhibitors and the IC_50_ are indicated at their targets.

The Jak/STAT3 pathway is a major signal pathway downstream of IL-6/IL-6R/gp130^26^. To verify the effect of IL-6 on STAT3 in PBL-1, we performed western blotting in the presence or absence of IL-6. While p-STAT3 was abundant in total cell lysates of PBL-1 in the presence of IL-6 (5 ng/mL), the dephosphorylation of p-STAT3 was induced within 1 h after the starvation of IL-6 (Fig. 5a). Moreover, the expression of total STAT3 decreased gradually after the starvation of IL-6. The phosphoinositide 3-kinase (PI3K)/Akt/mammalian target of rapamycin (mTOR) signalling pathway is another major pathway of IL-6 signalling downstream of IL-6R/gp130/Jak^29, 30^. Western blotting also revealed that IL-6 starvation induced the phosphorylation of eukaryotic translation initiation factor 4E (eIF4E) and p70S6K and the dephosphorylation of 4E-BP-1, which are factors downstream of the mTOR pathway (Fig. 5a). Phosphoinositide-dependent protein kinase-1 (PDK1), Akt, and p70S6K were phosphorylated at 1 to 6 hours after IL-6 starvation, but they were dephosphorylated immediately after phosphorylation. To compare the efficiency of drugs that affect the Jak/Stat3 and PI-3K/Akt/mTOR pathways with other pathways, we screened compound libraries containing more than 100 inhibitors for various oncogenic pathways. Inhibitors for Jak/STAT and PI3K/Akt/mTOR pathways induced cell death similar to general tyrosine kinase or cyclin dependent kinase inhibitors (Supplementary Table 3). The XTT assay revealed that the mTOR inhibitors, everolimus and rapamycin, also inhibited the mitochondrial metabolic activity of PBL-1 in a dose-dependent manner, and that the IC_50_ of each drug for PBL-1 was 187 pM and 9.74 nM, respectively (Fig. 5b and c). Everolimus was effective at a very low dose compared with the other compounds. These data suggested that the mTOR pathway has a significant effect on the growth of PBL-1.

### Therapeutic effects of drugs

**Figure 6:**
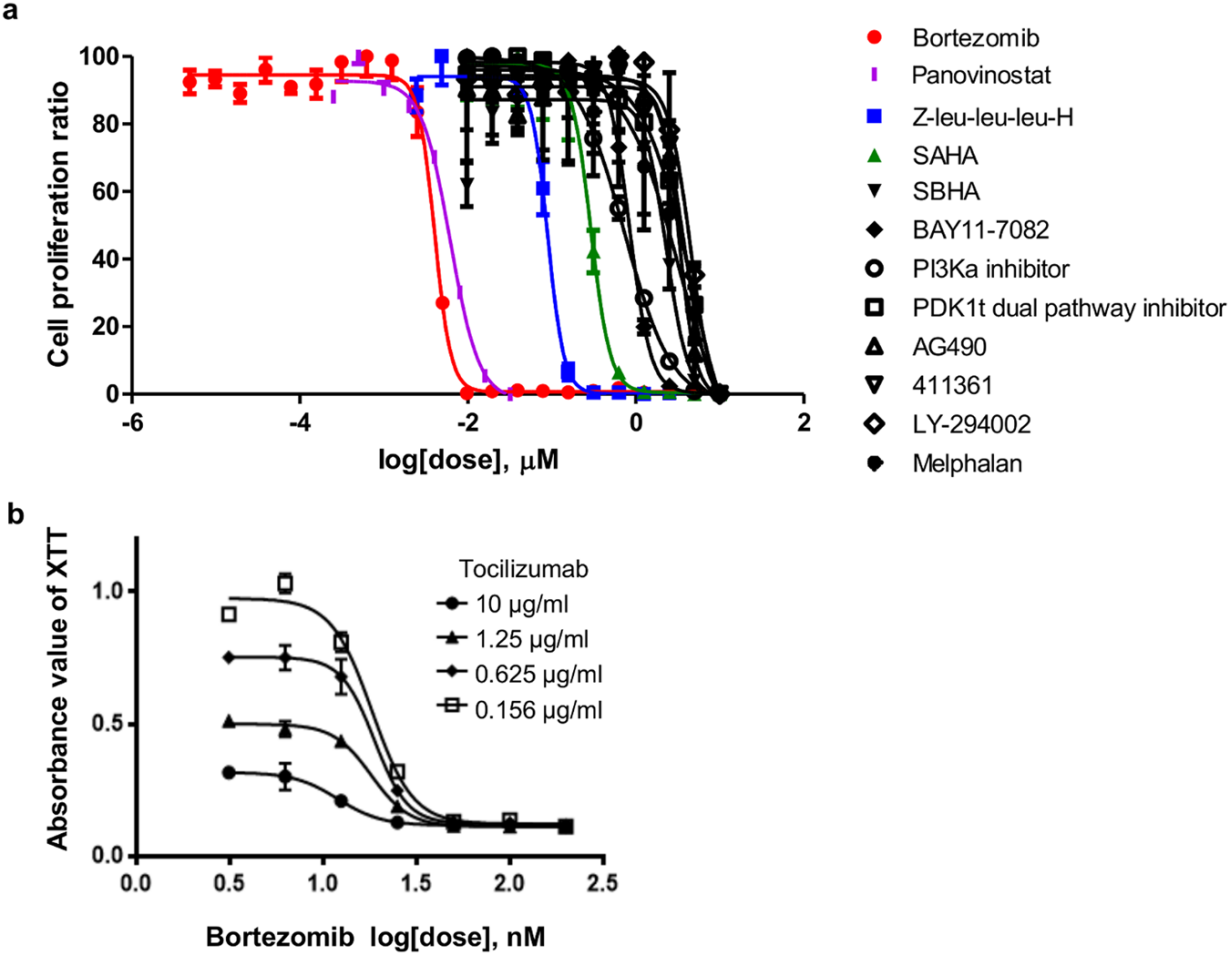
Therapeutic effects of drugs. (**a**) Effect of inhibitors on PBL-1 by XTT assay. Cell proliferation ratio (%) in XTT assay and the concentration of each drug are indicated on the vertical and horizontal axes. All inhibitors were added to medium supplemented with 5 ng/mL of IL-6. (**b**) Effects of combined tocilizumab and bortezomib on PBL-1. Tocilizumab and bortezomib were added to the medium simultaneously and the assay was performed after 48 hours incubation. The error bars in each panel represent the standard deviation of the measurements.

In addition to Jak, PI3K, Akt, and mTOR inhibitors, we tested other drugs that have an effect on malignant lymphoma or multiple myeloma *in vitro* and *in vivo* including proteasome inhibitors, melphalan, pomalidomide, and histone deacetylase (HDAC) inhibitors. The XTT assay showed that these drugs also inhibited the mitochondrial metabolic activity of PBL-1 in a dose-dependent manner (Fig. 6a, Supplementary Table 4). However, the IC_50_ of these drugs was higher than for the mTOR inhibitors. The combined therapeutic effect of the drugs was assessed using the Chou–Talalay Combination index (CI) method. The combination of bortezomib and tocilizumab (CI = 0.83, Fig. 6b), everolimus and tocilizumab (0.95), and panobinostat and tocilizumab (0.55) exhibited a synergistic effect (CI < 1), whereas antagonistic effects (CI > 1) were observed for three combinations (Supplementary Table 5).

## Discussion

The cell origin of PBL is thought to be the plasmablast^1, 31^, which is an activated B-cell in the germinal centre that has undergone somatic hypermutation and class switching recombination. The immunophenotype of plasmablasts is CD38 (+) MUM-1 (+) and CD20 (−), as reflected in PBL. The immunophenotype of PBL is similar to that of multiple myeloma except for those with EBV infection^32^. The established cell line, PBL-1, had an immunophenotype typical of plasmablasts, with morphological features that resembled plasmacytic tumours. To the best of our knowledge, there have been two previous reports of the establishment and characterization of cell lines from this distinct tumour^33, 34^, and one report of a xenograft model of EBV-negative-PBL^35^. A study of two EBV-positive cell lines, SPIBL-II and III, established by Friis *et al*. did not report the immunophenotype or culture method used including IL-6-dependency^33^. The SPIBL-II and III cell lines were established from the pleural effusion of an HIV-positive case of plasmablastic lymphoma. Considering that PBL occurred in the ascites as an effusion lymphoma in our case, such an effusion form of PBL that is very rare might contribute to the establishment of a cell line. Another two PBL cell lines, KY-1 and KY-2, reported by Matsuki *et al*. were established from a immunocompetent patient without HIV infection, and were CD20 (−), CD138 (+), VS38c (+), EMA (+), but CD38 (−) and EBV (−)^34^. IL-6-dependency in the culture medium was not described for these reported cell lines, although IL-6 might be associated with the establishment of these cell lines in a xenograft model^34, 35^.

The established PBL cell line in this report was IL-6 dependent. The dependency of cell activation by IL-6 through the gp130/Jak/STAT signalling pathway has been established in multiple myeloma^26, 27^. Starvation of IL-6 in PBL-1 led to the dephosphorylation of p-STAT3 and the phosphorylation of Akt, PDK1, and p70 within 1 h; however, p-Akt, p-PDK1, and p-p70 were dephosphorylated within 24 h (Fig. 5a). Although the factors upstream of the mTOR pathway were temporarily phosphorylated, they were dephosphorylated within a few hours. This phenomenon might be explained by negative feedback downstream of mTOR^36^. Inhibitors to factors upstream of mTOR did not dephosphorylate p-p70, but rapamycin and everolimus effectively dephosphorylated p-p70^37, 38^. mTOR is considered the bottleneck of the pathway. Tocilizumab is an antibody to soluble and membranous IL-6R, and inhibits IL-6 signal transduction^28^. Tocilizumab inhibited mitochondrial metabolic activity in PBL-1 in a dose-dependent manner (Fig. 4f), suggesting the inhibition of IL-6 signalling might be an effective treatment for PBL. *In vivo*, PBL cells require IL-6 secreted from surrounding cells such as macrophages. PBL can also secrete cytokines that act on surrounding cells; indeed, IL-8 expression was observed in the supernatant of PBL-1 (Supplementary Figure 1). Furthermore, an autocrine IL-6 mechanism might be involved in the growth of PBL. In primary effusion lymphoma, which is also considered to be derived from plasmablasts, KSHV-encoded viral IL-6 is secreted from lymphoma cells and induces the proliferation of the tumour by an autocrine mechanism^39–41^. The dependency of PBL on IL-6 secretion and the PBL morphology suggests PBL are biologically related to primary effusion lymphoma.

EBV infected PBL-1 show Latency I. The integration of EBV in host cells is often observed in tumours and cell lines^12^. Generally, integration by a virus occurs randomly at fragile sites in the host genome^42^. Although the integration site of EBV has not been identified in PBL-1, Southern blot analysis using an EBV-Bam-L probe (Fig. 3a) and dot-like signals of EBV observed in chromosomes by FISH (Fig. 3b), suggested that EBV did integrate into the host genome of PBL-1. Real-time PCR and droplet digital PCR analysis revealed that chemical and immunological stimulations did not induce the reactivation of EBV in PBL-1. In addition, a miRNA cluster in the BamHI-A Rightward Transcript (BART) region of the EBV genome was completely defective in PBL-1. Thus, miR-BARTs are not essential for maintaining this cell line. B95.8 also has a large deletion in BART DNA; however, no report has described deletion of the whole BART genome in EBV among any cases of lymphoma or EBV strains isolated from various diseases or healthy donors^43^. miR-BARTs were reported to have anti-apoptotic or oncogenic functions^44, 45^. A large deletion causing the complete lack of miR-BARTs might be associated with an abortive state. Only EBNA-1 and Qp promoters were expressed in PBL-1, whereas other EBV-encoded genes were not expressed. Recently, it was reported that EBNA-1 enhanced the expression of IL-6R^46, 47^ or had linkage with STAT regulation^48^, suggesting it might play a role in the survival of PBL-1.

In this study, bortezomib, pomalidomide, tocilizumab, and mTOR inhibitors (rapamycin, everolimus) were effective at inducing apoptosis in PBL-1. Everolimus, an mTOR inhibitor, is an immunosuppressive drug used for the treatment of breast cancer, neuroendocrine tumours, and kidney cancer; however, there has been no report on the therapeutic use of everolimus for PBL. Inhibitors of the mTOR pathway such as everolimus or rapamycin were more effective on PBL-1 than inhibitors to other factors downstream of IL-6 signalling such as the Jak/Stat pathway. This suggests that mTOR-associated factors play an important role in the IL-6 signalling pathway in PBL-1, and that IL-6 signalling pathways, especially mTOR, are a potential therapeutic target for PBL. Furthermore, in this study, bortezomib, a proteasome inhibitor used for the therapy of multiple myeloma, also exhibited a cytotoxic effect on PBL-1. This finding may indicate that PBL-1 has similar pharmacological responses to multiple myeloma. A combination of HDAC and proteasome inhibitors enhanced apoptosis in some lymphomas, multiple myeloma, EBV-associated tumours and cell lines^49–53^. These drugs might be potential therapeutic candidates for the treatment of PBL.

## Methods

### Patient

A 60-year-old Japanese male patient with HIV-1 infection was admitted with fever, weight loss, and ascites. His blood CD4 (+) cell counts were 316/μL. Flow cytometry analysis indicated the presence of CD19 (−), CD20 (−), CD38 (+) cells in the ascites. Positron emission transfer imaging revealed a large tumour in the transversus colon, and its biopsied specimen showed the infiltration of tumour cells similar to those in the ascites. Tumour cells were CD19 (−), CD20 (−), CD79a (−), CD38 (+), CD138 (+), CD30 (−), EBV- LMP-1 (−), EBV-EBNA-2 (−), KSHV-latency-associated nuclear antigen 1 (−), CD3 (−) CD5 (−), CD10 (−), BCL6 (−), MUM1 (+), and >95% Ki67 (MIB-1) (+) by immunohistochemistry, and EBER-1 (+) by *in situ* hybridization. These results led to a diagnosis of AIDS-related PBL. Six courses of CHOP chemotherapy induced a remission, but the patient eventually succumbed to the recurrence of disease.

### Cell culture

To establish a new cell line, tumour cells were collected from the patient ascites and cultured in RPMI 1640 culture medium supplemented with 10% patient ascites, 10% FBS, 10 ng/mL of insulin (Sigma-Aldrich, St. Louis, MO), and 10 ng/mL of transferrin (Sigma-Aldrich) at 37 °C with 5% CO_2_. Supplementation of insulin and transferrin was gradually reduced during passages. Finally, cells were grown in RPMI 1640 with 5 ng/mL of IL-6 (PeproTech, Rocky Hill, NJ) and 10% FBS. Single cell cloning was performed using a limiting dilution method in IL-6 containing medium. For the IL-6 starvation study, cells were centrifuged, washed twice with phosphate buffered saline (PBS), and cultured in RPMI 1640 supplemented with 10% FBS without IL-6. EBV-positive human cell lines (Raji and LCL), B95.8, an EBV-positive marmoset cell line, and EBV-negative cell lines (Bjab, BCBL-1, TY-1, PCM6, and Ramos) were cultured in RPMI 1640 medium with 10% FBS. Cells were stained with trypan blue and cell viability was measured using a TC10 automated cell counter (Bio-Rad, New York, NY).

### Immunofluorescence assay

Suspended cells were centrifuged onto glass slides via Cytospin (Thermo Fisher Scientific, Waltham, MA) to form a single layer of 5 × 10^4^ cells per spot. The samples were incubated with mouse monoclonal antibodies to CD20, CD38, CD138, and LMP-1 of EBV (Dako, Copenhagen, Denmark) diluted 1:100 with Block Ace (DS Pharma Biomedical, Osaka, Japan) for 1 h at room temperature, followed by Alexa Fluor 488-conjugated goat anti-mouse IgG (H+L) (Molecular Probes, Eugene, OR) diluted 1:500 with PBS for 1 h at room temperature. Nuclear staining was performed with 1 μg/mL 4′,6-diamidino-2-phenylindole. Images were captured with a fluorescence microscope (Olympus, Tokyo, Japan).

### Electron microscopy

PBL-1 cells were pelleted by centrifugation, fixed with 2.5% glutaraldehyde and 2% paraformaldehyde in 0.1 M phosphate buffer (pH 7.4) for 2 h at room temperature, post-fixed in 1% osmium tetroxide, and embedded in Epon. Ultrathin sections were stained with uranyl acetate and lead citrate, and observed under a transmission electron microscope (HT7700, Hitachi High Technologies, Tokyo, Japan) at 80 kV.

### RT-PCR

RT-PCR to detect EBV latency–associated transcripts was performed as described previously^54^.

### XTT assay

The viability of PBL-1 cells was examined using a XTT (tetrazolium derivative) assay kit (Roche Molecular Biochemicals, Indianapolis, IN). Cells were cultured in 96-well plates in triplicate at a density of 2 × 10^5^ cells/mL in RPMI 1640 medium containing 10% FBS in the presence or absence of 5 ng/mL IL-6 and/or applied drugs. After 48 h of culture, the XTT labelling mixture was added according to the manufacturer’s instructions. Colour development of formazan production was measured spectrophotometrically with Multiskan MS (LabSystems/Thermo Fisher Scientific) at 450 nm^55^. The cell proliferation ratio was calculated by the OD of a sample divided by the OD of the positive controls (IL-6-supplied PBL-1).

### Western blot analysis

Protein extraction and immunoblotting were performed as described previously^56^. Briefly, 1 × 10^6^ cells were lysed in 100 μL of M-PER lysis buffer containing Halt protease and phosphatase inhibitor cocktail (Pierce Biotechnology, Rockford, IL). Cell lysates were subjected to sodium dodecyl sulfate-polyacrylamide gel electrophoresis and transferred onto a polyvinylidene fluoride microporous membrane (Immobilon-P Transfer Membrane, Millipore, Bedford, MA) using the NuPAGE system (Life Technologies, Carlsbad, CA). The membranes were blocked with Block Ace and probed with primary antibodies followed by horse radish peroxidase-conjugated anti-mouse or anti-rabbit antibodies (Promega, Madison, WI) with an immunoreaction enhancer solution (Can Get Signal, Toyobo, Osaka, Japan). Blots were visualized by Super-Signal West Femto Chemiluminescent Substrate (Thermo Fisher Scientific) and images were captured with a C-Digit Blot Scanner (LI-COR Biosciences, Lincoln, NE).

### Anti-IL6R antibody and drug screening

Anti-IL6R antibody, tocilizumab (Actemra), was purchased from Chugai Pharmaceutical, Tokyo, Japan, and used at 48 ng–100 μg/mL. To identify drugs that inhibited PBL-1 cell proliferation, PBL-1 cells were cultured in RPMI 1640 with 10% FBS, 5 ng/mL of IL-6, and drugs from the InhibitorSelect 96-well Protein Kinase Inhibitor Library I, II, III (Calbiochem, San Diego, CA) in a 96-well plate at two final concentrations (10 nM and 1 μM). We also added 7 HDAC inhibitors (HDAC inhibitor Set II: Sigma Aldrich) and other antitumour drugs to PBL-1 cells with 5 ng/mL of IL-6 and cultured them for 48 h.

### Analysis for synergism and antagonism of drugs

PBL-1 cells were cultured in RPMI 1640 with 10% FBS, 5 ng/mL of IL-6 and two drugs (bortezomib, everolimus, tocilizumab and panobinostat) each added as a 2-fold dilution series from 6.4 μg/mL (tocilizumab), 32 nM (bortezomib and panobinostat) or 1.6 nM (everolimus) to determine the IC_50_. XTT assay was performed after 48 hr of culture. A constant combination ratio experiment for the 8 point assay was carried out, as the ratio of (IC_50_ of drug 1)/(IC_50_ of drug 2) was constant. The Chou–Talalay Combination index (CI) was calculated according to a study by Chou *et al*. using CompuSyn Version 1.0 software (http://www.combosyn.com/)^57^. A CI value of <1, = 1, or >1 indicates synergy, additivity, and antagonism, respectively.

### Statistical analysis

All results were expressed as the mean ± SD of data obtained in triplicate, from at least three individual experiments. All statistical analyses were performed with Excel software (Microsoft, Bellevue, WA), and GraphPad Prism Version 6.0 software (GraphPad Software, La Jolla, CA). P values < 0.05 were considered statistically significant.

### Ethical statement

The study protocol was approved by the Institutional Review Board, National Institute of Infectious Diseases (Approval No. 512) and Tokyo Metropolitan Komagome Hospital (Approval No. 1552). Informed consent was obtained from the patient with PBL. All methods were performed in accordance with the relevant guidelines and regulations.

Further methods are provided in the supplemental Materials and Methods.

### Data availability

The datasets generated during and/or analysed during the current study are available from the corresponding author on reasonable request.

## Electronic supplementary material

Supplementary information

## References

[1] Castillo JJ, Bibas M, Miranda RN (2015). The biology and treatment of plasmablastic lymphoma. Blood.

[2] Stein, H. H. N. & Campo, E. *In WHO Classification of Tumors of the Haematopoietic and Lymphoid Tissues*. 256–257 (IARC, 2008).

[3] Ota, Y. *et al*. Classification of AIDS-related lymphoma cases between 1987 and 2012 in Japan based on the WHO classification of lymphomas, fourth edition. *Cancer Med* **3**, 143–153 (2014). 10.1002/cam4.178 PMC3930399 24407967

[4] Carbone A (2002). AIDS-related non-Hodgkin’s lymphomas: from pathology and molecular pathogenesis to treatment. Hum Pathol.

[5] Morscio J (2014). Clinicopathologic comparison of plasmablastic lymphoma in HIV-positive, immunocompetent, and posttransplant patients: single-center series of 25 cases and meta-analysis of 277 reported cases. Am J Surg Pathol.

[6] Castillo J, Pantanowitz L, Dezube BJ (2008). HIV-associated plasmablastic lymphoma: lessons learned from 112 published cases. Am J Hematol.

[7] Swerdlow, S. H., C. E. & Harris, N. L. In *WHO classification of tumours of haematopoietic and lymphoid tissues* (IARC, 2008).

[8] Luo WJ (2004). Epstein-Barr virus is integrated between REL and BCL-11A in American Burkitt lymphoma cell line (NAB-2). Lab Invest.

[9] Takakuwa T, Luo WJ, Ham MF, Wada N, Aozasa K (2005). Identification of Epstein-Barr virus integrated sites in lymphoblastoid cell line (IB4). Virus Res.

[10] Gao J, Luo X, Tang K, Li X, Li G (2006). Epstein-Barr virus integrates frequently into chromosome 4q, 2q, 1q and 7q of Burkitt’s lymphoma cell line (Raji). J Virol Methods.

[11] Lestou VS (1993). Non-random integration of Epstein-Barr virus in lymphoblastoid cell lines. Genes Chromosomes Cancer.

[12] Hurley EA (1991). When Epstein-Barr virus persistently infects B-cell lines, it frequently integrates. J Virol.

[13] Popescu NC, Chen MC, Simpson S, Solinas S, DiPaolo JA (1993). A Burkitt lymphoma cell line with integrated Epstein-Barr virus at a stable chromosome modification site. Virology.

[14] Al-Malki MM, Castillo JJ, Sloan JM, Re A (2014). Hematopoietic cell transplantation for plasmablastic lymphoma: a review. Biol Blood Marrow Transplant.

[15] Barta SK (2013). Treatment factors affecting outcomes in HIV-associated non-Hodgkin lymphomas: a pooled analysis of 1546 patients. Blood.

[16] Bibas M, Castillo JJ (2014). Current knowledge on HIV-associated Plasmablastic Lymphoma. Mediterr J Hematol Infect Dis.

[17] Loghavi S (2015). Stage, age, and EBV status impact outcomes of plasmablastic lymphoma patients: a clinicopathologic analysis of 61 patients. J Hematol Oncol.

[18] Zelenetz AD (2010). NCCN Clinical Practice Guidelines in Oncology: non-Hodgkin’s lymphomas. J Natl Compr Canc Netw.

[19] Castillo JJ, Reagan JL, Sikov WM, Winer ES (2015). Bortezomib in combination with infusional dose-adjusted EPOCH for the treatment of plasmablastic lymphoma. Br J Haematol.

[20] Fernandez-Alvarez, R. *et al*. Bortezomib plus CHOP for the treatment of HIV-associated plasmablastic lymphoma: clinical experience in three patients. *Leuk Lymphoma*, 1–4 (2015). 10.3109/10428194.2015.1050666 25976108

[21] Elyamany G, Al Mussaed E, Alzahrani AM (2015). Plasmablastic Lymphoma: A Review of Current Knowledge and Future Directions. Adv Hematol.

[22] Hirosawa M, Morimoto H, Shibuya R, Shimajiri S, Tsukada J (2015). A striking response of plasmablastic lymphoma of the oral cavity to bortezomib: a case report. Biomark Res.

[23] Cencini E (2016). Long-term remission in a case of plasmablastic lymphoma treated with COMP (cyclophosphamide, liposomal doxorubicin, vincristine, prednisone) and bortezomib. Eur J Haematol.

[24] Fedele PL (2016). Infusional dose-adjusted epoch plus bortezomib for the treatment of plasmablastic lymphoma. Ann Hematol.

[25] Jones SA, Scheller J, Rose-John S (2011). Therapeutic strategies for the clinical blockade of IL-6/gp130 signaling. J Clin Invest.

[26] Dechow T (2014). GP130 activation induces myeloma and collaborates with MYC. J Clin Invest.

[27] Taga T, Kishimoto T (1995). Signaling mechanisms through cytokine receptors that share signal transducing receptor components. Curr Opin Immunol.

[28] Mihara M (2005). Tocilizumab inhibits signal transduction mediated by both mIL-6R and sIL-6R, but not by the receptors of other members of IL-6 cytokine family. Int Immunopharmacol.

[29] Shi Y, Hsu JH, Hu L, Gera J, Lichtenstein A (2002). Signal pathways involved in activation of p70S6K and phosphorylation of 4E-BP1 following exposure of multiple myeloma tumor cells to interleukin-6. J Biol Chem.

[30] Raje N (2004). Combination of the mTOR inhibitor rapamycin and CC-5013 has synergistic activity in multiple myeloma. Blood.

[31] Dolcetti R, Gloghini A, Caruso A, Carbone A (2016). A lymphomagenic role for HIV beyond immune suppression?. Blood.

[32] Vega F (2005). Plasmablastic lymphomas and plasmablastic plasma cell myelomas have nearly identical immunophenotypic profiles. Mod Pathol.

[33] Friis A (2013). Epstein Barr virus DNA analysis in blood predicts disease progression in a rare case of plasmablastic lymphoma with effusion. Infect Agent Cancer.

[34] Matsuki E (2011). Identification of loss of p16 expression and upregulation of MDR-1 as genetic events resulting from two novel chromosomal translocations found in a plasmablastic lymphoma of the uterus. Clin Cancer Res.

[35] Chapuy B (2016). Diffuse large B-cell lymphoma patient-derived xenograft models capture the molecular and biological heterogeneity of the disease. Blood.

[36] Manning BD (2004). Balancing Akt with S6K: implications for both metabolic diseases and tumorigenesis. J Cell Biol.

[37] Jefferies HB (1997). Rapamycin suppresses 5′TOP mRNA translation through inhibition of p70s6k. EMBO J.

[38] Guo H (2016). Everolimus exhibits anti-tumorigenic activity in obesity-induced ovarian cancer. Oncotarget.

[39] Meads MB, Medveczky PG (2004). Kaposi’s sarcoma-associated herpesvirus-encoded viral interleukin-6 is secreted and modified differently than human interleukin-6: evidence for a unique autocrine signaling mechanism. J Biol Chem.

[40] Rosean TR (2016). KSHV-encoded vIL-6 collaborates with deregulated c-Myc to drive plasmablastic neoplasms in mice. Blood Cancer J.

[41] Osborne J, Moore PS, Chang Y (1999). KSHV-encoded viral IL-6 activates multiple human IL-6 signaling pathways. Hum Immunol.

[42] Reisinger J, Rumpler S, Lion T, Ambros PF (2006). Visualization of episomal and integrated Epstein-Barr virus DNA by fiber fluorescence *in situ* hybridization. Int J Cancer.

[43] Palser AL (2015). Genome diversity of Epstein-Barr virus from multiple tumor types and normal infection. J Virol.

[44] Kang D, Skalsky RL, Cullen BR (2015). EBV BART MicroRNAs Target Multiple Pro-apoptotic Cellular Genes to Promote Epithelial Cell Survival. PLoS Pathog.

[45] Ross N, Gandhi MK, Nourse JP (2013). The Epstein-Barr virus microRNA BART11-5p targets the early B-cell transcription factor EBF1. Am J Blood Res.

[46] Tempera I (2015). Identification of MEF2B, EBF1, and IL6R as Direct Gene Targets of Epstein-Barr Virus (EBV) Nuclear Antigen 1 Critical for EBV-Infected B-Lymphocyte Survival. J Virol.

[47] Lu F (2010). Genome-wide analysis of host-chromosome binding sites for Epstein-Barr Virus Nuclear Antigen 1 (EBNA1). Virol J.

[48] Chen H (2001). Linkage between STAT regulation and Epstein-Barr virus gene expression in tumors. J Virol.

[49] Hui KF, Leung YY, Yeung PL, Middeldorp JM, Chiang AK (2014). Combination of SAHA and bortezomib up-regulates CDKN2A and CDKN1A and induces apoptosis of Epstein-Barr virus-positive Wp-restricted Burkitt lymphoma and lymphoblastoid cell lines. Br J Haematol.

[50] Bhatt S (2013). Efficacious proteasome/HDAC inhibitor combination therapy for primary effusion lymphoma. J Clin Invest.

[51] Hui KF, Lam BH, Ho DN, Tsao SW, Chiang AK (2013). Bortezomib and SAHA synergistically induce ROS-driven caspase-dependent apoptosis of nasopharyngeal carcinoma and block replication of Epstein-Barr virus. Mol Cancer Ther.

[52] Dimopoulos M (2013). Vorinostat or placebo in combination with bortezomib in patients with multiple myeloma (VANTAGE 088): a multicentre, randomised, double-blind study. Lancet Oncol.

[53] Dasmahapatra G (2010). The pan-HDAC inhibitor vorinostat potentiates the activity of the proteasome inhibitor carfilzomib in human DLBCL cells *in vitro* and *in vivo*. Blood.

[54] Iwasaki Y (1998). Establishment and characterization of a human Epstein-Barr virus-associated gastric carcinoma in SCID mice. J Virol.

[55] Cortes-Dericks L, Froment L, Kocher G, Schmid RA (2016). Human lung-derived mesenchymal stem cell-conditioned medium exerts *in vitro* antitumor effects in malignant pleural mesothelioma cell lines. Stem Cell Res Ther.

[56] Kanno T (2015). Fumagillin, a potent angiogenesis inhibitor, induces Kaposi sarcoma-associated herpesvirus replication in primary effusion lymphoma cells. Biochem Biophys Res Commun.

[57] Chou TC (2006). Theoretical basis, experimental design, and computerized simulation of synergism and antagonism in drug combination studies. Pharmacol Rev.

