# Supplementary material for: Interleukin-6-dependent growth in a newly established plasmablastic lymphoma cell line and its therapeutic targets
Source: Sci Rep. 2017 Aug 31;7:10188. doi: 10.1038/s41598-017-10684-5 (PMC5579229; doi:10.1038/s41598-017-10684-5)
Supplement: Supplementary file 1 — Supplementary information [file 41598_2017_10684_MOESM1_ESM.doc]

**Supplementary information**

Supplementary Methods

Supplementary Tables 1-7

Supplementary Figures 1-8

**Interleukin-6-dependent growth in a newly established plasmablastic lymphoma cell line and its therapeutic targets**

**Sohtaro Mine, Tsunekazu Hishima, Akihiko Suganuma, Hitomi Fukumoto, Yuko Sato, Michiyo Kataoka, Tsuyoshi Sekizuka, Makoto Kuroda, Tadaki Suzuki, Hideki Hasegawa, Masashi Fukayama, and Harutaka Katano**

**Supplementary Methods**

**Luminex**

Cytokines in ascites and culture medium were measured with Luminex according to the manufacturer’s instructions (Luminex, Austin, TX).

***In situ* hybridization for EBV-encoded small RNAs (EBER)**

*In situ* hybridization for EBER was performed as described previously^1^.

**Flow cytometry**

Cells were fixed and permeabilized using BD Cytofix fixation buffer and BD Phosflow perm buffer III (BD Biosciences Pharmingen, San Diego, CA). Fluorescein isothiocyanate-conjugated mouse monoclonal antibodies to CD3ε, CD4, CD8, CD20, CD30, CD38, CD45, CD45-RO, CD98, CD138, IgD, Ig κ, Ig λ were obtained from BD Biosciences Pharmingen.  Monoclonal antibodies used were LFA1 (BD Biosciences Pharmingen), MDM2, VCAM (Santa Cruz Biotechnology, Santa Cruz, CA), IgM (Life Technologies, Carlsbad, CA), gp80 (Thermo Fisher Scientific, Waltham, MA), gp130 and Blimp-1 (Cell Signaling Technology, Danvers, MA). Primary antibody incubation for 1 h was followed by incubation with Alexa Fluor 488-conjugated anti-mouse IgG (Molecular Probe, Eugene, OR) as a secondary antibody for 30 min. Cells were washed with stain buffer (BD Biosciences Pharmingen) three times and then analyzed by flow cytometry (Cyflow counter, Partec, Görlitz, Germany). Data were analyzed using FlowJo software (Flow Jo, Ashland, OR). For the Annexin V and propidium iodide (PI) apoptosis assay, cells were stained with MEBCYTO Apoptosis Kit (MBL, Nagoya, Japan) and analyzed by flow cytometry.

**PCR analysis and direct sequencing**

EBV, KSHV, and HIV DNA were amplified by conventional PCR in Supplementary Figure 3a^2^. A DNA fragment of EBV BNLF-1 with 320-bp length was amplified with the following primers: BNLF-1-forward (5ʹ–GCCAAAAGCTGCCAGATGGT-3ʹ) and BNLF-1-reverse (5ʹ-ACTGATGAGTAAGTATTACA-3ʹ). EBV type specific PCR was performed as described previously ^3^. For screening various virus infections, more than 160 virus genomes were examined with the multivirus real-time PCR system as described previously ^4^. Cluster regions of miR-BARTs and BHRFs in the EBV genome in Figure 2 were amplified with the primers listed in Supplementary Table 6. The IgH/c-myc genome containing c-Myc breakpoint was amplified, and the PCR product direct-sequenced^5, 6^. The sequence of c-Myc breakpoint was deposited in GenBank (accession no. LC164671). The IgH/c-myc genome in Supplementary Figure 4f was amplified with the following primers: PCR-1: Myc-5530r (5’-ATCCAGCGTCTAAGCAGCTGCA-3’) vs. Myc-5089f (5’-AGAGGCTTGGCGGGAAAAAGA-3’), PCR-2: IgH-960466f (5ʹ- GCACAGCAGCGAATCGTGAAAT-3ʹ) vs. IgH-960990r (5ʹ- TGGTCAAAACGCAGTCCCGCAT-3ʹ), PCR-3: Myc-5530r vs IgH-960990r. The c-myc, p53, and myd88 genomes were amplified as described previously ^7-9^.

**Genome walking to determine a deletion in the EBV genome**

Deletion in the EBV BART region was determined by genome walking using Universal GenomeWalker 2.0 (Clontech, Takara Bio Company, Mountain View, CA). Sequences of gene specific primers (GSP) were 5ʹ-ACTCCTGGTGGTACTCTGTGTCCTGT-3ʹ for GSP1 and 5ʹ-TGGACTTTTATGCCTGCTCCTCATCT-3ʹ for GSP2. To detect break points, the following primers were used in Figure 2; primer A: 5ʹ-AACTGAGGCATAAACAGGGCGT-3ʹ, primer B: 5ʹ-ACCTGAAGGTGACCAACATTGA-3ʹ, primer C: 5ʹ-TGGGAACACCTCCTTGTCGTAGA-3ʹ, and primer D: 5ʹ-TCTCGAGGCCCTCTGTGGCGAGA-3ʹ.

**Quantitative real-time PCR**

DNA and RNA samples were extracted using a DNeasy Blood & Tissue Kit and RNeasy Mini Kit (Qiagen, Hilden, Germany), respectively**.** EBV copies were analyzed by real-time PCR^10^. The DNA copy numbers per cell were calculated by dividing the EBV copy numbers by half of the beta-actin copy numbers, because each cell had two copies of the gene in two alleles. Real-time PCR was performed by Mx3005P (Agilent Technologies, Santa Clara, CA).

**Cytogenetic analysis**

Carnoy’s solution-fixed chromosome slides were prepared by a short-term culture method. In brief, cells were suspended in a petri dish filled with RPMI 1640 medium with 10% FBS and 0.01 μg/mL colcemid. The cells were incubated for 15 min in 0.075M KCl at 37°C and fixed in Carnoy’s solution. Karyotype analysis was carried out at different passages choosing 20 well-banded metaphases each time by Chromocenter (Tottori, Japan).

**Southern blot analysis**

Southern blot hybridization was performed to determine the IgH gene rearrangements and integration of the EBV genome, using an IgH joining region (JH) probe ^11^ and EBV–BamHI-L probe^12^. The standard method of Southern blotting was used for this analysis^13^. Southern blot analysis for JH was carried out by SRL (Tokyo, Japan).

**Immunoglobulin gene rearrangement analysis by BioMed2**

Immunoglobulin gene rearrangement was analyzed by the BioMed2 method as described previously ^14, 15^.

**Immunofixation electrophoresis (IFE)**

Forty-eight-hour culture supernatants from PBL-1 without FBS supplemented with 5 ng/mL IL-6 were collected by centrifugation. IFE with antisera to immunoglobulin A (IgA), IgM, IgG, κ, and λ for the supernatant was performed with the SPIFE ImmunoFix kit according to the manufacturer’s instructions (Helena Laboratories, Beaumont, TX) by LSI Medience (Tokyo, Japan) ^16^.

**Next Generation Sequencing (NGS)**

The TruSeq Small RNA-Seq Sample Prep Kit (Illumina, San Diego, CA) was used according to the manufacturer’s protocol to prepare a small RNA library for sequencing. The quality and yield after sample preparation was measured with a Bioanalyzer with a High Sensitivity DNA kit (Agilent Technologies) and corresponded to the expected 150 bp. DNA sequencing was accomplished using Miseq (Illumina) using a MiSeq reagent kit v3 according to the manufacturer’s protocol. Sequence reads were analyzed with CLC Genomics Workbench (version 9.0, Qiagen). After adaptor trimming, less than 15 nucleotide lengths and more than 26 nucleotide lengths of reads were removed, and all reads with a 15-25 nucleotide length were analyzed against miRBase release 21 retrieved from the miRNA database (http://www.mirbase.org/). Homo_sapiens.GRCh37.57.ncrna was used as the comprehensive non-coding RNA database (http://www.ncrna.org/). All annotated reads matched to pre-miRNA were counted as miRNA reads. Sequence data of small RNAs analyzed by NGS in this study were deposited in the DNA Data Bank of Japan (DDBJ; accession number: DRA004825; Bioproject PRJDB4936).

**Fluorescent in situ hybridization (FISH)**

FISH was carried out as described previously ^17-19^. In brief, the EBV-specific probes used were PCR products from PBL-1 DNA with Biotin-11-dUTP (Roche Molecular Biochemicals, Indianapolis, IN) as shown in Supplementary Table 7. Hybridization was performed as described previously ^17-19^. Carnoy’s solution-fixed chromosome slides were prepared in the same way as for the cytogenetic analysis, and were rinsed for 5 min in 2× SSC (1× SSC contains 0.15 M NaCl and 0.015 M sodium citrate) and then dehydrated with 70 and 95% ethanol before air-drying. For DNA-specific hybridization, RNase A (100 μg/mL in 2× SSC) treatment was performed at 37°C for 1 h, followed by pepsin treatment (0.02 μg/mL in 10 mM HCl) at 37°C before hybridization. Slides were denatured for 3 min in 70% formamide/2× SSC at 70°C. Preparations were dehydrated with cold 70, 90, and 100% ethanol for 3 min each and air-dried. Then, 100 ng of probe and 5 μg of human placental DNA were suspended in 100 μL of deionized formamide and heated at 99°C for 3 min. An equal volume of hybridization buffer was added, so that the final hybridization solution contained 50% formamide, 2× SSC, and 10% dextran sulfate. The slides were incubated at 37°C overnight. Biotin-labeled DNA was detected by Alexa-fluor 488 or Rhodamine-conjugated streptavidin (Molecular Probe). Chromosomes and nuclei were counterstained with propidium iodide or 4′,6-diamidino-2-phenylindole. The slides were examined with a fluorescence microscope (Olympus, Tokyo, Japan).

**Droplet digital PCR**

Droplet digital PCR was performed using a QX100 droplet digital PCR system (Bio-Rad, Pleasanton, CA) as described previously^20^. The ddPCR reaction mixture consisted of 10 μL of a 2× ddPCR supermix for probes (Bio-Rad), 900 nM (final) of primers, 250 nM (final) probes, and 1 ng of sample nucleic acid in a final volume of 20 μL. Sequences of the probe and primer set were as follows: EBNA1 F (5ʹ-TACAGGACCTGGAAATGGCC-3ʹ), EBNA1 R (5ʹ-TCTTTGAGGTCCACTGCCG-3ʹ) primers and EBNA1 fam-Probe (5ʹ-(6FAM)AGGGAGACACATCTGGACCAGAAGGC(BHQ1)-3ʹ) for EBNA1^21^; beta-actin-Fwd (5′-TGAGCGCGGCTACAGCTT-3′), beta-actin-Rev (5′-TCCTTAATGTCACGCACGATTT-3′) primers and beta-actin-HEX-probe (5′-HEX-ACCACCACGGCCGAGCGG-BHQ1-3′) for beta-actin^22^. The mixture was loaded into a DG8 cartridge, (Bio-Rad) with 70 μL of droplet generation oil (Bio-Rad), and droplets were formed in a droplet generator (Bio-Rad). After processing, the droplets were transferred to 200-μL PCR tubes, and PCR amplification was carried out on a thermal cycler GeneAmp PCR system 9700 (Applied Biosystems, Foster City, CA) using a thermal profile beginning at 95°C for 10 min, followed by 40 cycles of 94°C for 30 s and 60°C for 60 s, 1 cycle of 98°C for 10 min, and ending at 4°C. After amplification, the plate was loaded onto the droplet reader (Bio-Rad). Data were analyzed with QuantaSoft analysis software (Bio-Rad). The quantification measurements of the target molecule were presented as the copy numbers per μL of DNA sample.

**Drug screening**

Cells were cultured in RPMI 1640 with drugs from the InhibitorSelect 96-well Protein Kinase Inhibitor Library I, II, III (Calbiochem, San Diego, CA) and 5 ng/ml IL-6 in a 96-well plate at two final concentrations (10 nM and 1 μM). Absorbance values of the XTT assay were listed. Abbreviations of Human Kinome Branch were described in the manufacturer’s protocol.

**Chemicals, cytokines, and antibodies**

Everolimus (07741), sodium butyrate (B5887), melphalan (M2011), Diethyl (bromodifluoromethyl) phosphonate (411361), LY-294002 hydrochloride (L9908), AG490 (T3434), Bay 11-7082 (B5556), IC261 (I0658), pomalidomide (P0018), HDAC inhibitor set II (EPI009) containing panobinostat, SAHA, SBHA, and Cl994 were purchased from Sigma-Aldrich (St. Louis, MO). Z-VAD-FMK (FMK001) was from R&D systems (Minneapolis, MN). Bortezomib (sc-217785) was from Santa Cruz Biotechnology. Rapamycin (ab120224) was from Abcam (Cambridge, UK). Z-leu-leu-leu-H was obtained from Peptide Institute (Osaka, Japan).

Recombinant human IL-6 (rhIL-6, 200-06), rhIFN-β (300-02BC), rhRANTES (300-06), rhGM-CSF (300-03), rhIFNc (300-02), rhIL-8 (200-08), rhVEGF165 (100-20), rhHGF (1 00-39), rhIL-1 (200-01RA), rhIL-15 (200-15), rhIP-10/CXCL10 (300-12), rhMCP-1/CCL2 (300-04), rhMIG/CXCL9 (300-26), rhIL-12 (200-12), rhIL-4 (200-04), rhIL-7 (200-07), rhIL-6Ra (200-06R), rhIL-11 (300-11), rhIL-10 (200-10), and rhIL-13 (200-13) were purchased from PeproTec EC Ltd., (London, UK). Rh TNF-α and oncostatin M were obtained from R&D Systems.

The following antibodies were used in western blotting: Akt (pan, #4691), p-Akt (Thr308, #2965), Blimp-1/PRDI-BF1 (#9115), Caspase-3 (#9662), Caspase-8 (#9746), Caspase-9 (#9502), STAT3 (12H6, #9139), p-STAT3 (Ser727, #9136), mTOR (#2983), p-mTOR (Ser2481, #2974), mTOR Regulation Antibody Sampler Kit, p-PDK1 (Ser241, #3438), p-PI3 Kinase p85 (Tyr 458)/p55 (Tyr199, #4228), p-p70 (Thr389, #9234), eIF4E (C46H6, #2067), p-eIF4E (Ser209, #9741), 4E-BP1 (53H11, #9644), p-4E-BP-1 (The37/46, #2855), MYD88 (D80F5, #4283), and XBP-1s (D2C1F, #12782) were obtained from Cell Signaling Technology. Mcl-1 (S-19) and GAPDH (FL-335, sc-25778) were from Santa Cruz Biotechnology Inc. PI3-Kinase (610045), Bcl-2 Related Sampler Kit (612742), Apoptosis Sampler Kit I (612741), and Apoptosis Sampler Kit II (612743) were from BD Transduction Laboratories (Franklin Lakes, NJ).

**Measurement of sIL-6R concentrations**

The concentration of sIL-6Ra in the 48-hr culture supernatant of PBL-1 was measured with Quantikine colorimetric sandwich ELISA according to the manufacturer’s instructions (R&D Systems).

**Preparation of Lentiviral Vectors and Transduction of PBL-1**

Lentivirus vectors and packaging plasmids were provided from Riken BioResource Center (Tsukuba, Japan). The packaging construct pCAG-HIVgp vector, in which all accessory genes (vif, vpr, vpu, and nef) and regulatory genes (tat and rev) were deleted, was used in this study for the preparation of lentiviral vectors ^23, 24^. The expression of IL-6 or control genes (IFN-α and -β) were amplified by PCR (primers used were as follows: for IL-6 :IL-6-F63-*Not*1 (5ʹ-CTCGCGGCCGCatgaactccttctccacaag-3ʹ) and IL-6-R701-*Bam*H1 (5ʹ-ACAGGATCCctacatttgccgaagagccc-3ʹ), for IFN-β: IFNβ-F75-*Not*1 (5ʹ-CTCGCGGCCGCatgaccaacaagtgtctcct-3ʹ) and (IFNβ-R638-*Bam*H1 5ʹ-ACAGGATCCtcagtttcggaggtaacctg-3ʹ), for IFN-α2: IFNα2-F69-*Not*1 (5ʹ-CTCGCGGCCGCatggccttgacctttgcttt-3ʹ) and IFNα2-R635-*Bam*H1 (5ʹ-ACAGGATCCtcattccttacttcttaaac-3ʹ) and inserted into the multiple cloning sites of the pCSII-EF-MCS-internal ribosome entry site 2 (IRES2)-Venus. Lentiviral vectors pseudotyped with the vesicular stomatitis Indiana virus G glycoprotein were generated as previously described ^25^. In brief, 293 T cells were transiently co-transfected with appropriate amounts of the SIN vector construct (pCSII-EF-[IL-6, IFN-α2, or IFN-β]-IRES2-Venus), the packaging construct (pCAG-HIVgp) and the vesicular stomatitis virus G glycoprotein–expressing construct (pCMV-VSV-G-RSV-Rev). The viral supernatants were collected 72 h after transfection and concentrated 1000-fold by ultracentrifugation at 19,400 rpm (SW28; Beckman Coulter, Brea, CA) for 2 h and virus precipitates was resuspended. Vector titers were determined by infection of 293 cells with serial dilutions of the vector stocks, followed by counting Venus expressing cells. For infection, 10^5^ PBL-1 cells were cultured in RPMI 1640 medium without IL-6 in the presence or absence of 10% FBS containing lentivirus or retrovirus supernatants at a multiplicity of infection of 100 in 96-well U-bottom plates for 72 h at 37°C in 5% CO_2_.

**BrdU assay**

The proliferation of PBL-1 cells was examined using a Cell Proliferation ELISA, BrdU (colorimetric) assay kit (Roche Molecular Biochemicals, Indianapolis, IN) according to the manufacturer’s instructions.

**References**

1. Howe JG, Steitz JA. Localization of Epstein-Barr virus-encoded small RNAs by in situ hybridization. *Proc Natl Acad Sci U S A* 1986; **83**: 9006-10.

2. Katano H, Hoshino Y, Morishita Y, Nakamura T, Satoh H, Iwamoto A, et al. Establishing and characterizing a CD30-positive cell line harboring HHV- 8 from a primary effusion lymphoma. *J Med Virol* 1999; **58**: 394-401.

3. Hassan R, White LR, Stefanoff CG, de Oliveira DE, Felisbino FE, Klumb CE, et al. Epstein-Barr virus (EBV) detection and typing by PCR: a contribution to diagnostic screening of EBV-positive Burkitt's lymphoma. *Diagn Pathol* 2006; **1**: 17.

4. Katano H, Kano M, Nakamura T, Kanno T, Asanuma H, Sata T. A novel real-time PCR system for simultaneous detection of human viruses in clinical samples from patients with uncertain diagnoses. *J Med Virol* 2011; **83**: 322-30.

5. Busch K, Borkhardt A, Wossmann W, Reiter A, Harbott J. Combined polymerase chain reaction methods to detect c-myc/IgH rearrangement in childhood Burkitt's lymphoma for minimal residual disease analysis. *Haematologica* 2004; **89**: 818-25.

6. Basso K, Frascella E, Zanesco L, Rosolen A. Improved long-distance polymerase chain reaction for the detection of t(8;14)(q24;q32) in Burkitt's lymphomas. *Am J Pathol* 1999; **155**: 1479-85.

7. Katano H, Sato Y, Sata T. Expression of p53 and human herpesvirus 8 (HHV-8)-encoded latency-associated nuclear antigen (LANA) with inhibition of apoptosis in HHV-8-associated malignancies. *Cancer* 2001; **92**: 3076-84.

8. Oishi N, Kondo T, Nakazawa T, Mochizuki K, Tanioka F, Oyama T, et al. High prevalence of the MYD88 mutation in testicular lymphoma: Immunohistochemical and genetic analyses. *Pathol Int* 2015; **65**: 528-35.

9. de Souza CR, Leal MF, Calcagno DQ, Costa Sozinho EK, Borges Bdo N, Montenegro RC, et al. MYC deregulation in gastric cancer and its clinicopathological implications. *PLoS One* 2013; **8**: e64420.

10. Dehee A, Asselot C, Piolot T, Jacomet C, Rozenbaum W, Vidaud M, et al. Quantification of Epstein-Barr virus load in peripheral blood of human immunodeficiency virus-infected patients using real-time PCR. *J Med Virol* 2001; **65**: 543-52.

11. Beishuizen A, Verhoeven MA, Mol EJ, Breit TM, Wolvers-Tettero IL, van Dongen JJ. Detection of immunoglobulin heavy-chain gene rearrangements by Southern blot analysis: recommendations for optimal results. *Leukemia* 1993; **7**: 2045-53.

12. Luo WJ, Takakuwa T, Ham MF, Wada N, Liu A, Fujita S, et al. Epstein-Barr virus is integrated between REL and BCL-11A in American Burkitt lymphoma cell line (NAB-2). *Lab Invest* 2004; **84**: 1193-9.

13. Southern EM. Detection of specific sequences among DNA fragments separated by gel electrophoresis. *J Mol Biol* 1975; **98**: 503-17.

14. Langerak AW, Groenen PJ, Bruggemann M, Beldjord K, Bellan C, Bonello L, et al. EuroClonality/BIOMED-2 guidelines for interpretation and reporting of Ig/TCR clonality testing in suspected lymphoproliferations. *Leukemia* 2012; **26**: 2159-71.

15. van Dongen JJ, Langerak AW, Bruggemann M, Evans PA, Hummel M, Lavender FL, et al. Design and standardization of PCR primers and protocols for detection of clonal immunoglobulin and T-cell receptor gene recombinations in suspect lymphoproliferations: report of the BIOMED-2 Concerted Action BMH4-CT98-3936. *Leukemia* 2003; **17**: 2257-317.

16. Weiss BM, Abadie J, Verma P, Howard RS, Kuehl WM. A monoclonal gammopathy precedes multiple myeloma in most patients. *Blood* 2009; **113**: 5418-22.

17. Popescu NC, Chen MC, Simpson S, Solinas S, DiPaolo JA. A Burkitt lymphoma cell line with integrated Epstein-Barr virus at a stable chromosome modification site. *Virology* 1993; **195**: 248-51.

18. Szeles A, Falk KI, Imreh S, Klein G. Visualization of alternative Epstein-Barr virus expression programs by fluorescent in situ hybridization at the cell level. *J Virol* 1999; **73**: 5064-9.

19. Wolf J, Jox A, Skarbek H, Pukrop T, Bartnitzke S, Pawlita M, et al. Selective loss of integrated Epstein-Barr virus genomes after long-term cultivation of Burkitt's lymphoma x B-lymphoblastoid cell hybrids due to chromatin instability at the integration site. *Virology* 1995; **212**: 179-85.

20. Hayden RT, Gu Z, Ingersoll J, Abdul-Ali D, Shi L, Pounds S, et al. Comparison of droplet digital PCR to real-time PCR for quantitative detection of cytomegalovirus. *J Clin Microbiol* 2013; **51**: 540-6.

21. Ryan JL, Fan H, Glaser SL, Schichman SA, Raab-Traub N, Gulley ML. Epstein-Barr virus quantitation by real-time PCR targeting multiple gene segments: a novel approach to screen for the virus in paraffin-embedded tissue and plasma. *J Mol Diagn* 2004; **6**: 378-85.

22. Kuramochi H, Hayashi K, Uchida K, Miyakura S, Shimizu D, Vallbohmer D, et al. Vascular endothelial growth factor messenger RNA expression level is preserved in liver metastases compared with corresponding primary colorectal cancer. *Clin Cancer Res* 2006; **12**: 29-33.

23. Miyoshi H. Gene delivery to hematopoietic stem cells using lentiviral vectors. *Methods Mol Biol* 2004; **246**: 429-38.

24. Shibuya K, Shirakawa J, Kameyama T, Honda S, Tahara-Hanaoka S, Miyamoto A, et al. CD226 (DNAM-1) is involved in lymphocyte function-associated antigen 1 costimulatory signal for naive T cell differentiation and proliferation. *J Exp Med* 2003; **198**: 1829-39.

25. Tahara-Hanaoka S, Sudo K, Ema H, Miyoshi H, Nakauchi H. Lentiviral vector-mediated transduction of murine CD34(-) hematopoietic stem cells. *Exp Hematol* 2002; **30**: 11-7.

**Supplementary Table 1.** Results of PBL-1 by multivirus real-time PCR analysis.

| Positive |
| --- |
| EBV  Human beta-actin-DNA (Internal control)  Human beta 2 microglobulin-RNA (Internal control) |
| Negative (under detection limit) |
| Polyomavirus: JC virus, BK virus, Simian virus 40  Papillomavirus: human papillomavirus 6, 11, 16, 18, 31, 33, 35, 39, 45, 51, 52,56,58,59, 66, 68, 73  Parvovirus: Adeno-associated virus 1,2,3,5, Parvovirus B19, human bocavirus, adenovirus A, B, C, D,E, F  Herpes virus: Human herpesvirus 1-3, 5-8, B virus  Poxvirus: Variola virus, Monkey pox virus, Molluscum contagiosum virus  Anellovirus: Torque teno virus  Hepadnavirus: Hepatitis B virus  Other: Mimivirus  Filovirus: Ebola virus, Marburg virus  Bunyavirus: Crimean-Congo Hemorrhagic Fever virus, Hemorrhagic fever with renal syndrome virus (Hantaan, Dovrava, Puumala, and Seoul), Rift valley fever virus, Sin Nombre virus  Arenavirus: Lassa virus, Junin, Guanarito, Machupo, Sabia  Togavirus: Equine encephalitis virus (Venezuelan, Eastern, and Western), Sindbis virus, Mayaro virus, Getah virus, Chikungunya virus, Rubella virus  Enterovirus: Enterovirus 68, 71, Poliovirus 1,2,3, Coxsackievirus A2, A3, A4, A5, A6, A8, A9, A10, A16, A21, A24, B1, B2, B3, B4, B5, B6, Echovirus 5, 6, 7, 9, 11, 13, 14, 16, 17, 18, 25, 30, Parechovirus 1,3, Rhinovirus A, B, rotavirus, reovirus 1-4, Melaka virus, Colorado tick borne fever virus  Flavivirus: Dengue virus 1,2, Japanese encephalitis virus, Murray Valley encephalitis virus, St. Louis encephalitis virus, West Nile virus, Tick-borne encephalitis virus, Yellow fever virus  Orthomyxovirus: Influenza virus A, B, C, H5N1,  Paramyxovirus: Parainfluenza virus 1-3, Hendra virus, Mumps virus, Measles virus, Sendai virus, RS virus A,B, metapneumovirus, Nipah virus  Rabdovirus: Rabies virus, Lyssavirus 5, 6, Chandipura virus, Duvenhage virus  Coronavirus: Coronavirus OC43, 229E, NL63, SARS virus  Calicivirus: Sapovirus, Norwalk-like virus 1, 2  Hepatitis virus: Hepatitis A virus, Hepatitis C virus, Hepatitis D virus, Hepatitis E virus, GB virus  Retrovirus: human immunodeficiency virus 1, human T cell leukemia virus 1,2  Other: Astrovirus, Borna disease virus |

**Supplementary Table 2.** Counts of read sequences by NGS.

| **Samples** | **PBL-1** | | **Original tumor of PBL-1** | |
| --- | --- | --- | --- | --- |
| **Annotation** | **Count** | **Percentage** | **Count** | **Percentage** |
| Annotated | 1,381,414 | 65.40 % | 977,011 | 68.50% |
| - Homo sapiens | 484,194 | 100.00 % | 699,911 | 100.00% |
| - EBV | 2* | 0.00 % | 1* | 0.00% |
| Unannotated | 731,396 | 34.60 % | 449,465 | 31.50% |
| Total Reads | 2,112,810 | 100.00 % | 1,426,476 | 100.00% |

*miR-BHRF1

**Supplementary Table 3.** Results of drug screening. OD values in XTT assay are shown.

| **Product Description** | **Human Kinome Branch** | **PBL1(1μM)** | **PBL1(10nM)** |
| --- | --- | --- | --- |
| Akt Inhibitor IV | **AGC** | 0.0877 | 1.1327 |
| PDK1/Akt/Flt Dual Pathway Inhibitor | **AGC, ATYPICAL, TK** | 0.0894 | 0.9706 |
| K-252a, Nocardiopsis sp. | **AGC** | 0.0894 | 1.1355 |
| PDGF Receptor Tyrosine Kinase Inhibitor IV | **TK** | 0.0904 | 0.9842 |
| eEF-2 Kinase Inhibitor, NH125 | Atypical | 0.091 | 1.341 |
| Cdk4 Inhibitor III | **CMGC** | 0.0915 | 1.3535 |
| TX-1918 | Atypical | 0.0932 | 0.9557 |
| Alsterpaullone, 2-Cyanoethyl | **CMGC** | 0.0937 | 0.9441 |
| Staurosporine, Streptomyces sp. | **AGC, CAMK, TK** | 0.0937 | 0.2682 |
| IKK Inhibitor VII | Other | 0.0937 | 1.6051 |
| BAY 11-7082 | **OTHER** | 0.0943 | 1.4738 |
| Cdk4 Inhibitor | **CMGC** | 0.0943 | 1.6627 |
| Fascaplysin, Synthetic | **CMGC** | 0.0943 | 1.5574 |
| IKK-2 Inhibitor V | Other | 0.0954 | 1.5772 |
| Cdk1 Inhibitor, CGP74514A | **CMGC** | 0.097 | 1.3727 |
| Staurosporine, Streptomyces sp. | **AGC, CAMK, TK** | 0.0976 | 0.224 |
| Chelerythrine Chloride | **AGC** | 0.0981 | 1.4751 |
| Keratinocyte Differentiation Inducer | CMGC | 0.0981 | 1.8373 |
| Akt Inhibitor X | **AGC** | 0.0992 | 1.0302 |
| Cdk1/2 Inhibitor III | **CMGC** | 0.0992 | 0.3375 |
| CR8, (S)-Isomer | CMGC | 0.0998 | 1.1304 |
| PKR Inhibitor | **OTHER** | 0.1014 | 1.356 |
| CR8, (R)-Isomer | CMGC | 0.102 | 1.0866 |
| Syk Inhibitor III | **TK** | 0.1025 | 1.4284 |
| Cdk2/9 Inhibitor | CMGC | 0.1025 | 1.0267 |
| Ro-31-8220 | AGC CMGC | 0.1025 | 1.0554 |
| Cdc7/Cdk9 Inhibitor | CMGC | 0.1031 | 1.1639 |
| Aurora Kinase/Cdk Inhibitor | **OTHER, CMGC** | 0.1047 | 1.5215 |
| PI 3-Kα Inhibitor VIII | Other | 0.1058 | 0.2082 |
| IKK-2 Inhibitor XI | Other | 0.1063 | 1.4168 |
| 5-Iodotubercidin | Other | 0.1063 | 1.7838 |
| Cdk/Crk Inhibitor | **CMGC** | 0.1069 | 0.6801 |
| UCN-01 | AGC CAMK CMGC TK | 0.108 | 0.7419 |
| PI 3-Kα Inhibitor IV | Other | 0.1091 | 1.6817 |
| IKK-2 Inhibitor VI | Other | 0.1107 | 0.9589 |
| SB 218078 | **CAMK** | 0.1135 | 0.9149 |
| IKK-2 Inhibitor IV | **OTHER** | 0.1173 | 1.2666 |
| PI-103 | **LIPID** | 0.1179 | 0.4917 |
| Indirubin Derivative E804 | **CMGC, TK** | 0.119 | 0.9658 |
| Wee1 Inhibitor | Other | 0.12 | 0.8656 |
| Akt Inhibitor VIII, Isozyme-Selective, Akti-1/2 | **AGC** | 0.1206 | 0.8846 |
| JAK Inhibitor I | **TK** | 0.1206 | 1.0475 |
| Herbimycin A, Streptomyces sp. | **TK** | 0.1228 | 0.6908 |
| JAK3 Inhibitor VI | **TK** | 0.1233 | 0.9465 |
| Olomoucine II | CMGC | 0.1233 | 1.8428 |
| EGFR Inhibitor | **TK** | 0.1244 | 1.3778 |
| p21-Activated Kinase Inhibitor III, IPA-3 | STE | 0.1255 | 1.779 |
| SU11652 | **TK** | 0.1261 | 1.1395 |
| Wee1/Chk1 Inhibitor | Other CAMK | 0.1261 | 0.8361 |
| Reversine | STE Other | 0.1299 | 1.2692 |
| SU9516 | **CMGC** | 0.1343 | 1.0736 |
| Bisindolylmaleimide I | **AGC** | 0.1354 | 1.0792 |
| JNK Inhibitor IX | **CMGC** | 0.1354 | 1.0703 |
| Compound 56 | **TK** | 0.1398 | 1.4344 |
| MK2a Inhibitor | **CAMK** | 0.1403 | 1.6936 |
| Staurosporine, N-benzoyl- | **AGC, CMGC, TK** | 0.142 | 0.9321 |
| Flt-3 Inhibitor II | **TK** | 0.1447 | 1.3854 |
| ATM/ATR Kinase Inhibitor | **ATYPICAL** | 0.1458 | 1.1112 |
| Cdk1 Inhibitor | **CMGC** | 0.1464 | 1.5256 |
| IC261 | **CK1** | 0.1655 | 1.5081 |
| GSK-3 Inhibitor IX | **CMGC** | 0.1738 | 1.1764 |
| Flt-3 Inhibitor III | **TK** | 0.1787 | 1.279 |
| Rapamycin | **AGC** | 0.1803 | 0.2986 |
| Gö 7874, Hydrochloride | AGC | 0.1809 | 1.9003 |
| SB220025 | **CMGC** | 0.199 | 0.8739 |
| Cdk2 Inhibitor IV, NU6140 | **CMGC** | 0.2215 | 1.3903 |
| Bisindolylmaleimide III, Hydrochloride | AGC | 0.2264 | 1.0036 |
| Alsterpaullone | **CMGC** | 0.2324 | 1.092 |
| PDGF Receptor Tyrosine Kinase Inhibitor III | **TK** | 0.2363 | 1.4329 |
| 1-Azakenpaullone | CMGC | 0.2434 | 1.0937 |
| Met Kinase Inhibitor | **TK** | 0.2593 | 1.1267 |
| p38 MAP Kinase Inhibitor III | **CMGC** | 0.2615 | 1.5098 |
| LY 294002, 4'-NH_2_ | Other | 0.2615 | 1.515 |
| Indirubin-3′-monoxime | **CMGC** | 0.2823 | 1.417 |
| EGFR/ErbB-2/ErbB-4 Inhibitor | **TK** | 0.2872 | 1.3733 |
| Wee1 Inhibitor II | Other | 0.2955 | 1.014 |
| Cdk1 Inhibitor IV, RO-3306 | CMGC | 0.2966 | 0.9613 |
| PIKfyve Inhibitor | Other | 0.2966 | 1.3091 |
| Aminopurvalanol A | **CMGC** | 0.3081 | 1.0168 |
| GSK3b Inhibitor XII, TWS119 | **CMGC** | 0.3103 | 1.2917 |
| Chk2 Inhibitor | CAMK | 0.3125 | 1.4655 |
| LY 294002 | **LIPID** | 0.347 | 1.2006 |
| SU6656 | **TK** | 0.3519 | 1.8114 |
| Aurora Kinase Inhibitor II | **OTHER** | 0.3547 | 0.9676 |
| VEGFR Tyrosine Kinase Inhibitor IV | **TK** | 0.3547 | 0.865 |
| Cdk Inhibitor, p35 | CMGC | 0.3563 | 1.5939 |
| GSK-3 Inhibitor XIII | **CMGC** | 0.3596 | 1.5599 |
| KN-93 | **CAMK** | 0.3601 | 1.3853 |
| PDGF RTK Inhibitor | **TK** | 0.3623 | 1.1237 |
| Cdk2 Inhibitor III | **CMGC** | 0.376 | 1.331 |
| Kenpaullone | **CMGC, CK1, TK** | 0.4084 | 1.4989 |
| AG 1024 | **TK** | 0.4188 | 1.1154 |
| NF-kB Activation Inhibitor | **OTHER** | 0.4221 | 0.7453 |
| Quercetagetin | CAMK | 0.4347 | 1.3291 |
| Purvalanol A | **CMGC** | 0.4457 | 0.9734 |
| MEK Inhibitor I | **STE** | 0.4583 | 1.0895 |
| Scytonemin, *Lyngbya* sp. | AGC CAMK CMGC Other | 0.4725 | 1.2118 |
| JNK Inhibitor V | **CMGC** | 0.4775 | 1.031 |
| Cdk9 Inhibitor II | CMGC | 0.4791 | 1.2461 |
| AMPK Inhibitor, Compound C | **CAMK** | 0.4813 | 1.0502 |
| Rho Kinase Inhibitor | AGC | 0.4879 | 1.2748 |
| Isogranulatimide | **CAMK, CMGC, ATYPICAL** | 0.4917 | 1.5206 |
| Polo-like Kinase Inhibitor II, BTO-1 | Other | 0.5235 | 0.998 |
| PDGF Receptor Tyrosine Kinase Inhibitor II | **TK** | 0.5547 | 1.3484 |
| PI 3-Kγ/CKII Inhibitor | Other CMGC | 0.558 | 1.5764 |
| Rho Kinase Inhibitor IV | **AGC** | 0.5717 | 1.1787 |
| PKCbII/EGFR Inhibitor | **TK, AGC** | 0.5761 | 1.1192 |
| KN-62 | **CAMKII** | 0.5822 | 1.1497 |
| PKCb Inhibitor | **AGC** | 0.5833 | 1.4811 |
| Sphingosine Kinase Inhibitor | **LIPID** | 0.5843 | 0.8622 |
| Roscovitine, (S)-Isomer | CMGC | 0.5882 | 0.9182 |
| Roscovitine | CMGC | 0.5909 | 1.077 |
| AGL 2043 | **TK** | 0.603 | 1.1131 |
| TGF-b RI Inhibitor III | **TKL** | 0.6085 | 1.2149 |
| Akt Inhibitor XII, Isozyme-Selective, Akti-2 | AGC | 0.6123 | 1.353 |
| H-89, Dihydrochloride | **AGC, CK1, CAMK** | 0.615 | 1.1605 |
| Indirubin-3′-monoxime, 5-Iodo- | CMGC | 0.6326 | 1.8644 |
| Rho Kinase Inhibitor V | AGC | 0.6507 | 1.1631 |
| GSK-3 Inhibitor X | **CMGC** | 0.6545 | 1.0594 |
| PIM1/2 Kinase Inhibitor V | CAMK | 0.6732 | 1.199 |
| STO-609 | **CAMK** | 0.677 | 0.9842 |
| MEK1/2 Inhibitor | **STE** | 0.6869 | 1.0352 |
| Bohemine | **CMGC** | 0.6874 | 1.5014 |
| MNK1 Inhibitor | **CAMK** | 0.6907 | 1.0786 |
| Adenosine Kinase Inhibitor | Other | 0.7055 | 1.2669 |
| MEK Inhibitor II | **STE** | 0.711 | 1.1689 |
| Cdk1/5 Inhibitor | **CMGC** | 0.7274 | 1.6259 |
| AG 490 | **TK** | 0.7417 | 1.3205 |
| Bisindolylmaleimide V | AGC | 0.7472 | 1.0507 |
| PD 169316 | **CMGC** | 0.7504 | 1.2349 |
| PIM1/2 Kinase Inhibitor VI | CAMK | 0.751 | 1.8396 |
| IGF-1R Inhibitor II | **TK** | 0.7554 | 1.4397 |
| Aurora Kinase Inhibitor III | **OTHER** | 0.757 | 1.0661 |
| Tpl2 Kinase Inhibitor | **CMGC** | 0.7587 | 1.0878 |
| Casein Kinase I Inhibitor, D4476 | **CK1, CMGC, TKL** | 0.7669 | 1.6134 |
| SB 239063 | CMGC | 0.7713 | 1.1791 |
| PP1 Analog II, 1NM-PP1 | **TK** | 0.7773 | 1.3808 |
| Gö 6976 | **AGC** | 0.7811 | 1.1335 |
| RSK Inhibitor, SL0101 | AGC | 0.7954 | 1.089 |
| PI 3-Kg Inhibitor | **LIPID** | 0.7987 | 1.135 |
| Syk Inhibitor II | **TK** | 0.7998 | 1.1908 |
| Tpl2 Kinase Inhibitor II | STE | 0.7998 | 0.9366 |
| SKF-86002 | **CMGC** | 0.8064 | 0.9057 |
| GSK-3b Inhibitor XI | **CMGC** | 0.8096 | 1.0043 |
| CaMKII Inhibitor, CK59 | CAMK | 0.8102 | 1.0427 |
| Lck Inhibitor | **TK** | 0.8113 | 1.2443 |
| PD 98059 | **STE** | 0.8179 | 1.3811 |
| SB 203580, Sulfone | CMGC | 0.8206 | 1.0411 |
| IKK Inhibitor X | Other | 0.8239 | 1.3291 |
| p38 MAP Kinase Inhibitor VIII | STE | 0.8332 | 1.3339 |
| PD 158780 | **TK** | 0.8354 | 1.5113 |
| Aurora Kinase Inhibitor III | **OTHER** | 0.842 | 1.0098 |
| Rho Kinase Inhibitor II | AGC | 0.8442 | 1.1328 |
| Ras/Rac Transformation Blocker, SCH 51344 | Other | 0.8497 | 1.4471 |
| Arcyriaflavin A, Synthetic | CMGC | 0.8727 | 1.2796 |
| p38 MAP Kinase Inhibitor | **CMGC** | 0.8776 | 1.3869 |
| ERK Inhibitor III | **CMGC** | 0.8787 | 1.1822 |
| Casein Kinase II Inhibitor III, TBCA | **CK1** | 0.8804 | 1.2959 |
| Bisindolylmaleimide IV | **AGC** | 0.8897 | 1.546 |
| Aloisine A, RP107 | **CMGC** | 0.8902 | 0.9667 |
| WHI-P180, Hydrochloride | CMGC | 0.9034 | 0.9326 |
| Akt Inhibitor V, Triciribine | **AGC** | 0.9072 | 0.9268 |
| JNK Inhibitor VIII | **CMGC** | 0.9094 | 1.4964 |
| Necrostatin-1 | TKL | 0.9094 | 1.2006 |
| Aloisine, RP106 | **CMGC** | 0.91 | 1.1371 |
| Compound 401 | Atypical | 0.9122 | 1.7296 |
| PKR Inhibitor, Negative Control | **OTHER** | 0.9176 | 1.1463 |
| ROCK Inhibitor, Y-27632 | **AGC** | 0.9204 | 1.6877 |
| Bcr-abl Inhibitor | **TK** | 0.9231 | 1.0573 |
| 4-Cyano-3-methylisoquinoline | AGC | 0.9231 | 1.3714 |
| ERK Inhibitor II, FR180204 | **CMGC** | 0.9275 | 1.6326 |
| AG 1296 | **TK** | 0.9324 | 1.2511 |
| SB 202190 | **CMGC** | 0.9324 | 1.1981 |
| PI 3-Kb Inhibitor II | **LIPID** | 0.9335 | 1.1569 |
| JNK Inhibitor II | **CMGC** | 0.9352 | 1.1931 |
| LY 303511 | **LIPID** | 0.9363 | 1.1855 |
| PD 174265 | **TK** | 0.9423 | 1.4035 |
| PIM1 Kinase Inhibitor IV | CAMK | 0.9439 | 1.0969 |
| DNA-PK Inhibitor II | **ATYPICAL** | 0.9489 | 1.3967 |
| ATM Kinase Inhibitor | **ATYPICAL** | 0.9533 | 1.1898 |
| Casein Kinase II Inhibitor I | CMGC | 0.956 | 1.3402 |
| DNA-PK Inhibitor V | **ATYPICAL** | 0.9637 | 0.9253 |
| SB 202474, Negative control for p38 MAPK inhibition studies | **INACTIVE** | 0.9648 | 1.1338 |
| Cdk2 Inhibitor II | CMGC | 0.967 | 1.9075 |
| Polo-like Kinase Inhibitor I | Other | 0.9692 | 1.199 |
| GSK-3b Inhibitor I | **CMGC, AGC, TK** | 0.9708 | 1.163 |
| VEGF Receptor 2 Kinase Inhibitor III | **TK** | 0.9801 | 0.9276 |
| cFMS Receptor Tyrosine Kinase Inhibitor | **TK** | 0.9851 | 1.2602 |
| Ste11 MAPKKK Activation Inhibitor | TK | 0.9894 | 1.0714 |
| SC-68376 | **CMGC** | 0.9905 | 0.9984 |
| Src Kinase Inhibitor I | **TK** | 0.9911 | 0.954 |
| JNK Inhibitor, Negative Control | **CMGC** | 0.9916 | 1.1162 |
| IKK-2 Inhibitor VIII | Other | 1.0021 | 1.4695 |
| Rho Kinase Inhibitor III, Rockout | **AGC** | 1.0026 | 0.8839 |
| AG 1295 | **TK** | 1.0075 | 1.2293 |
| MEK1/2 Inhibitor II | STE | 1.0103 | 1.6155 |
| HA1077, Dihydrochloride Fasudil | **AGC** | 1.0158 | 1.0561 |
| Diacylglycerol Kinase Inhibitor II | **LIPID** | 1.018 | 1.3235 |
| VEGF Receptor Tyrosine Kinase Inhibitor II | **TK** | 1.0185 | 0.9367 |
| AG 1478 | **TK** | 1.0223 | 0.9359 |
| DMBI | **TK** | 1.0262 | 1.2587 |
| Cdk4 Inhibitor II, NSC 625987 | **CMGC** | 1.0344 | 1.5106 |
| Casein Kinase II Inhibitor IV | CMGC | 1.0355 | 1.8436 |
| ML-7, Hydrochloride | CAMK | 1.0366 | 1.1855 |
| AG 9 | **TK** | 1.0388 | 0.9857 |
| AG 112 | **TK** | 1.0404 | 0.9653 |
| JAK3 Inhibitor II | **TK** | 1.0432 | 1.1184 |
| SB 203580 | **CMGC** | 1.0437 | 1.0987 |
| VEGF Receptor 2 Kinase Inhibitor II | **TK** | 1.0443 | 0.8914 |
| Compound 52 | **CMGC** | 1.0476 | 1.2065 |
| VEGF Receptor 2 Kinase Inhibitor IV | **TK** | 1.0514 | 0.954 |
| KT5720 | AGC | 1.0541 | 1.7264 |
| GSK-3 Inhibitor IX, Control, MeBIO | CMGC | 1.0558 | 1.8293 |
| PI 3-Kγ Inhibitor VII | Other | 1.0563 | 1.373 |
| EGFR/ErbB-2 Inhibitor | **TK** | 1.0569 | 1.2089 |
| PP3 | **TK** | 1.0635 | 1.2745 |
| Flt-3 Inhibitor | **TK** | 1.0651 | 1.3243 |
| PI 3-Kβ Inhibitor VI, TGX-221 | Other | 1.0651 | 1.4783 |
| Gö 6983 | **AGC** | 1.0772 | 1.4834 |
| TGF-b RI Kinase Inhibitor | **TKL** | 1.0799 | 1.2496 |
| BPIQ-I | **TK** | 1.0837 | 1.2707 |
| Syk Inhibitor | **TK** | 1.0837 | 1.4178 |
| VEGF Receptor 2 Kinase Inhibitor I | **TK** | 1.0848 | 0.9291 |
| GSK-3b Inhibitor VIII | **CMGC** | 1.0898 | 1.2324 |
| GTP-14564 | **TK** | 1.0963 | 1.1893 |
| GSK-3b Inhibitor II | **CMGC** | 1.0963 | 1.2374 |
| Cdk2/5 Inhibitor | CMGC | 1.0974 | 1.8173 |
| IKK-3 Inhibitor IX | Other | 1.0991 | 1.3682 |
| p38 MAP Kinase Inhibitor IV | STE | 1.1002 | 1.732 |
| Cdc2-Like Kinase Inhibitor, TG003 | **CMGC** | 1.1084 | 1.585 |
| IRAK-1/4 Inhibitor | **TKL** | 1.1133 | 1.2956 |
| JAK3 Inhibitor IV | **TK** | 1.127 | 1.4231 |
| MK-2 Inhibitor III | STE | 1.138 | 1.4958 |
| p38 MAP Kinase Inhibitor VII, SD-169 | STE | 1.138 | 1.6442 |
| ERK Inhibitor II, Negative control | **CMGC** | 1.144 | 1.6702 |
| Stem-Cell Factor/c-Kit Inhibitor, ISCK03 | TK | 1.1468 | 1.1009 |
| PIM1 Kinase Inhibitor II | CAMK | 1.1621 | 1.412 |
| KN-92 | NA | 1.1928 | 1.507 |
| DNA-PK Inhibitor III | **ATYPICAL** | 1.2016 | 1.3024 |
| p38 MAP Kinase Inhibitor VI, JX401 | STE | 1.2263 | 1.6027 |
| H-8, Dihydrochloride | AGC | 1.2465 | 1.8899 |
| Chk2 Inhibitor II | **CAMK** | 1.2739 | 1.3819 |
| IP3K Inhibitor | Other | 1.3014 | 1.6139 |
| HA 1004, Dihydrochloride | AGC | 1.3118 | 1.7958 |
| No drug | - | 1.0240 | 1.0761 |

**Supplementary Table 4**. The IC50 of drugs to PBL-1.

| Drug | Category | IC50 |
| --- | --- | --- |
| Everolimus | mTOR inhibitor | 187 pM |
| Bortezomib | Proteasome inhibitor | 4.03 nM |
| Panobinostat | HDAC inhibitor | 6.19 nM |
| Z-leu-leu-leu-H | Proteasome inhibitor | 8.92 nM |
| Rapamycin | mTOR inhibitor | 9.74 nM |
| Tocilizumab | Anti-IL-6R antibody | 41.3 nM |
| IC261 | Casein kinase inhibitor | 139 nM |
| SAHA | HDAC inhibitor | 291 nM |
| PI3Ka inhibitor | PI3K inhibitor | 725 nM |
| BAY11-7082 | NF-kB inhibitor | 843 nM |
| Cl994 | HDAC inhibitor | 1.35 μM |
| SBHA | HDAC inhibitor | 2.31 μM |
| AG490 | JAK inhibitor | 3.30 μM |
| Diethyl (bromodifluoromethyl) phosphonate | Stat3 inhibitor | 3.78 μM |
| LY-294002 | PI3K inhibitor | 4.49 μM |
| PDK1/Akt/Flt dual inhibitor | PDK1/Akt/Flt dual inhibitor | 4.57 μM |
| Pomalidomide | Immunomodulatory drug | 26.56 μM |

**Supplementary Table 5**. Chou–Talalay Combination index (CI) for drug combination treatment.

| Combination | CI |
| --- | --- |
| Bortezomib + Everolimus | 1.85 |
| Bortezomib + Tocilizumab | 0.83 |
| Tocilizumab + Everolimus | 0.95 |
| Everolimus + Panobinostat | 8.98 |
| Tocilizumab + Panobinostat | 0.55 |
| Bortezomib + Panobinostat | 37.2 |

**Supplementary Table 6.** PCR primers for miRNA clusters in the EBV genome.

| Product | Target | Strand | Primer name | Start | Sequence | Size of product |
| --- | --- | --- | --- | --- | --- | --- |
| E1 | miR-BARTs3,4,1 | forward | EBV139081F | 139,081 | tccctgtaaacacacaccac | 440 |
|  |  | reverse | EBV139520R | 139,520 | ttctacatcatgcctggttc |  |
| E2 | miR-BARTs15,5,16,17,6 | forward | EBV139501F | 139,501 | gaaccaggcatgatgtagaa | 690 |
|  |  | reverse | EBV140190R | 140,190 | tttagatctgtggttacatg |  |
| E3 | miR-BARTs21,18 | forward | EBV145451F | 145,451 | ttagatgttagctttgtgtt | 590 |
|  |  | reverse | EBV146040R | 146,040 | ggcccaaaccttcgcagcag |  |
| E4 | miR-BARTs7 | forward | EBV145911F | 145,911 | ttgttgccgttgaaagacgg | 610 |
|  |  | reverse | EBV146520R | 146,520 | tggccacactaaacacacaa |  |
| E5 | miR-BARTs8,9 | forward | EBV146701F | 146,701 | ttatttgggttacaagacct | 350 |
|  |  | reverse | EBV147050R | 147,050 | cacaatgaaacccaaagccc |  |
| E6 | miR-BARTs22, 10,11 | forward | EBV147131F | 147,131 | cggttgtcacaggtgctaga | 500 |
|  |  | reverse | EBV147630R | 147,630 | cgtgaaaggcactccagaat |  |
| E7 | miR-BARTs12,19,20 | forward | EBV147871F | 147,871 | acctaagacccgcccatcac | 550 |
|  |  | reverse | EBV148420R | 148,420 | ccaaaggacccgggatcacg |  |
| E8 | miR-BARTs13, 14 | forward | EBV148461F | 148,461 | catcttgacgttggaatgtc | 360 |
|  |  | reverse | EBV148820R | 148,820 | ctcctgggttggcgtttccg |  |
| E9 | miR-BARTs2 | forward | EBV152651F | 152,651 | gcagcaaaagaggaacttgc | 350 |
|  |  | reverse | EBV153000R | 153,000 | ggcaaagatccccagcggag |  |
| E10 | miR-BHRF1-1 | forward | EBV41581F | 41,581 | cctcaccatgacacactaag | 260 |
|  |  | reverse | EBV41840R | 41,840 | ccagatgcacccaacagccc |  |
| E11 | miR-BHRF1-2,3 | forward | EBV42991F | 42,991 | gggtgacacagtgcccatgc | 330 |
|  |  | reverse | EBV43320R | 43,320 | acactcacctcagttatttc |  |

**Supplementary Table 7**. PCR primers to produce FISH probes.

| Product | Target | Strand | Primer name | Start | Sequence | Size of product |
| --- | --- | --- | --- | --- | --- | --- |
| E10 | miR-BHRF1-1 | forward | EBV41581F | 41,581 | cctcaccatgacacactaag | 260 |
|  |  | reverse | EBV41840R | 41,840 | ccagatgcacccaacagccc |  |
| E11 | miR-BHRF1-2,3 | forward | EBV42991F | 42,991 | gggtgacacagtgcccatgc | 330 |
|  |  | reverse | EBV43320R | 43,320 | acactcacctcagttatttc |  |
| E23 | BBRF3 | forward | EBV107588F | 107,588 | aacagcttttactttatggt | 344 |
|  |  | reverse | EBV107931R | 107,931 | agttccaccgtctccgagacag |  |
| E24 | BBRF1 | forward | EBV103242F | 103,242 | agttgcggcagaagccgcgtctg | 253 |
|  |  | reverse | EBV103494R | 103,494 | acgctgcccctgattatcaacca |  |
| E25 | BKRF2 | forward | EBV99253F | 99,253 | aacagtcttcaggtctgacacc | 298 |
|  |  | reverse | EBV99550R | 99,550 | tacagccatggccaattgtagtt |  |
| E26 | EBNA1/BKRF1 | forward | EBV97385F | 97,385 | attgtctgttatttcatggtct | 286 |
|  |  | reverse | EBV97670R | 97,670 | tacgattgagggcgtctcctaa |  |
| E27 | EBNA3B/BERF2a | forward | EBV85722F | 85,722 | atacaggttatggggcaaggg | 249 |
|  |  | reverse | EBV85970R | 85,970 | tccgcactccagagtctgct |  |
| E31 | BBLF1 | forward | EBV108822F | 108,822 | aggaccacgggtgtcaccagca | 259 |
|  |  | reverse | EBV109080R | 109,080 | tcccagtcaacatcttcatcaa |  |
| E36 | BGLF3-2 | forward | EBV109223F | 109,223 | aagattccaggcctcatcctg | 259 |
|  |  | reverse | EBV109481R | 109,481 | ggagtcacagggagactttat |  |
| E37 | BGLF3-3 | forward | EBV109622F | 109,622 | aaaggcaacaggtcccccaaag | 279 |
|  |  | reverse | EBV109900R | 109,900 | gtctcctcgttagtccaagaga |  |
| E38 | BGLF3-4 | forward | EBV110021F | 110,021 | agtctccggactgcggaggaa | 254 |
|  |  | reverse | EBV110274R | 110,274 | tccgtgcgtggatgtcacggcg |  |
| E39 | BGLF3-5 | forward | EBV110422F | 110,422 | attttgtgggtggagtcagcgag | 272 |
|  |  | reverse | EBV110693R | 110,693 | aacaagatgctggatgtgcggct |  |

**Supplementary Figures**


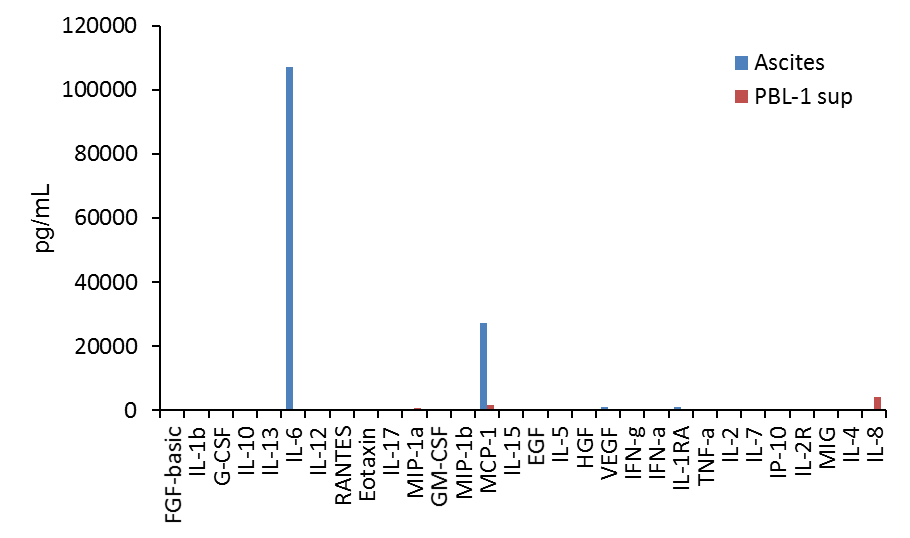


**Supplementary Figure 1**. Cytokines in the ascites and supernatant of primary cultures assessed by Luminex. Blue bars indicate cytokine levels in the ascites from the patient, and red bars indicate those in culture supernatants from PBL-1 without the addition of IL-6.


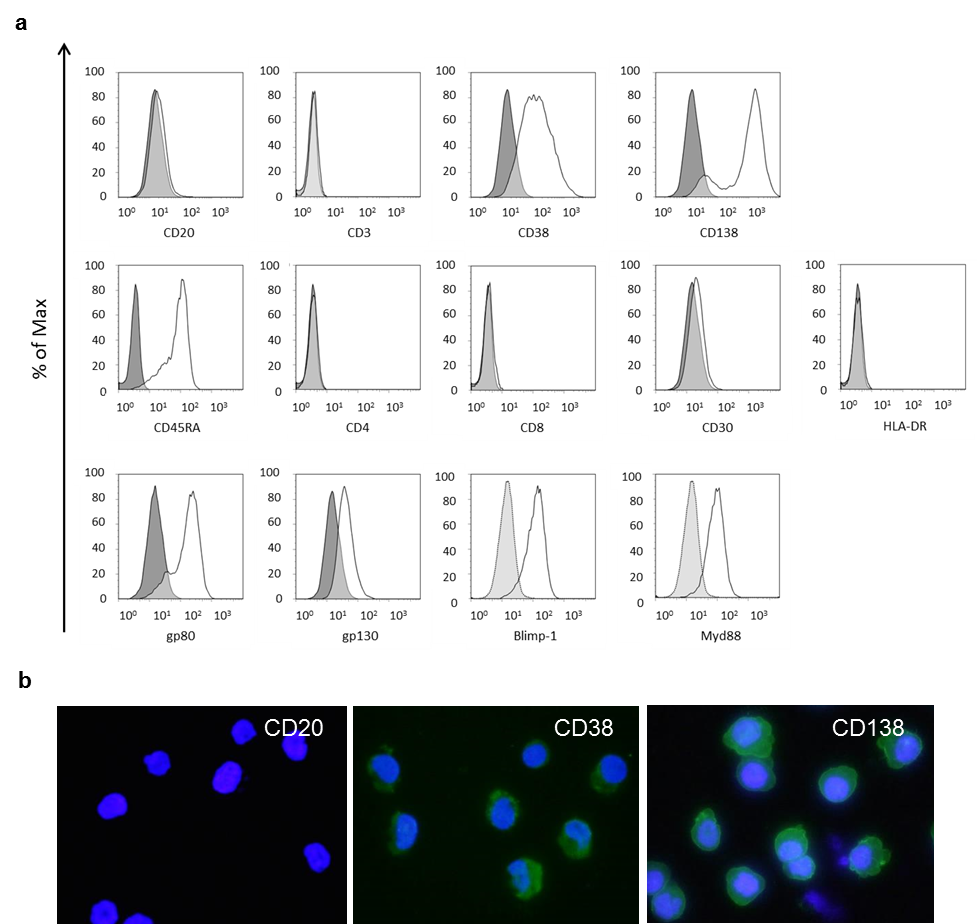


**Supplementary Figure 2.** Immunophenotypes of PBL-1. (a) Immunophenotypes of PBL-1 by flow cytometry. Gray peaks indicate negative control antibody. (b) Immunofluorescence assay of PBL-1. Green signals are specific for CD20, CD38, and CD138 antigens. Nuclei were counterstained with 4′,6-diamidino-2-phenylindole (blue). Human plasma cells were examined as a positive control, and showed positive signals in CD38 and CD138 (data not shown).


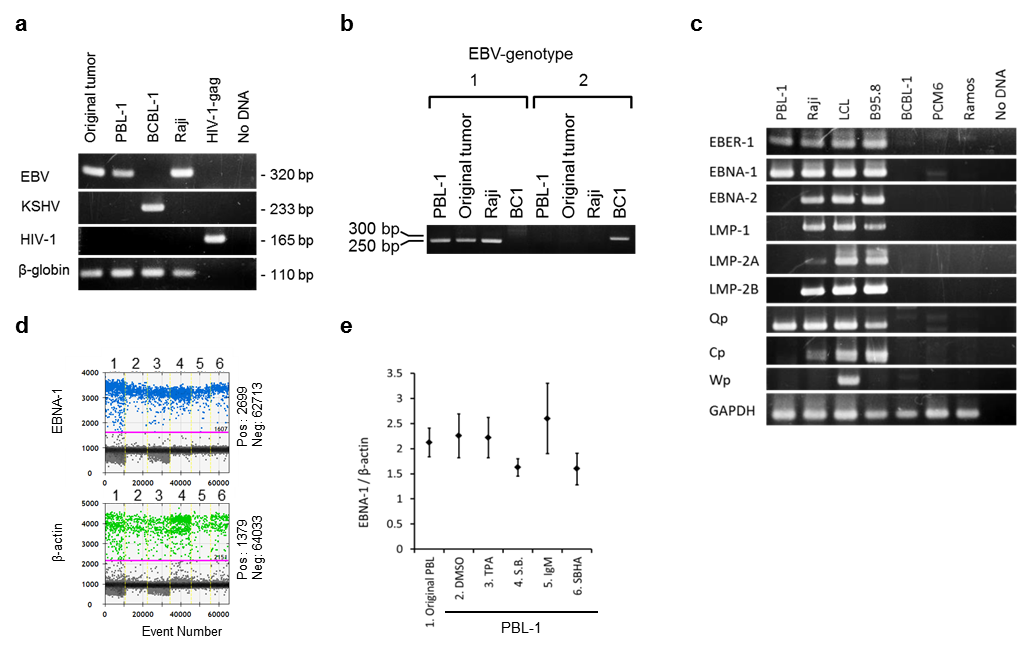


**Supplementary Figure 3.** EBV infection in PBL-1. (a) PCR analysis for EBV, KSHV, and HIV-1 in PBL-1. PCR products were electrophoresed in a 2% ME agarose gel. Original PBL indicates a DNA sample extracted from the primary ascites cells of the patient. HIV-gag is a plasmid encoding HIV-1 gag. (b) EBV genotype in PBL-1. PCR specific for EBV genotypes was performed on PBL-1 cell line, original tumor, Raji (positive control for genotype 1), and BC1 (positive control for genotype 2). (c) RT-PCR for EBV-encoded latency-associated transcripts and promoters. GAPDH mRNA was amplified as an internal control. (d and e) Digital PCR analysis for EBNA-1 and beta-actin DNA in PBL-1. 1: Primary cells of the patient used to generate PBL-1, 2: PBL-1 with DMSO (control), 3: PBL-1 with TPA, 4: PBL-1 with sodium butyrate, 5: PBL-1 with IgM, and 6: PBL-1 with SBHA. Droplet images of EBNA-1 and beta-actin DNA are shown with the average numbers of positive and negative droplets (e). Ratio of EBNA-1 /beta-actin in each sample is shown with an error bar of 68% confidence intervals (f).


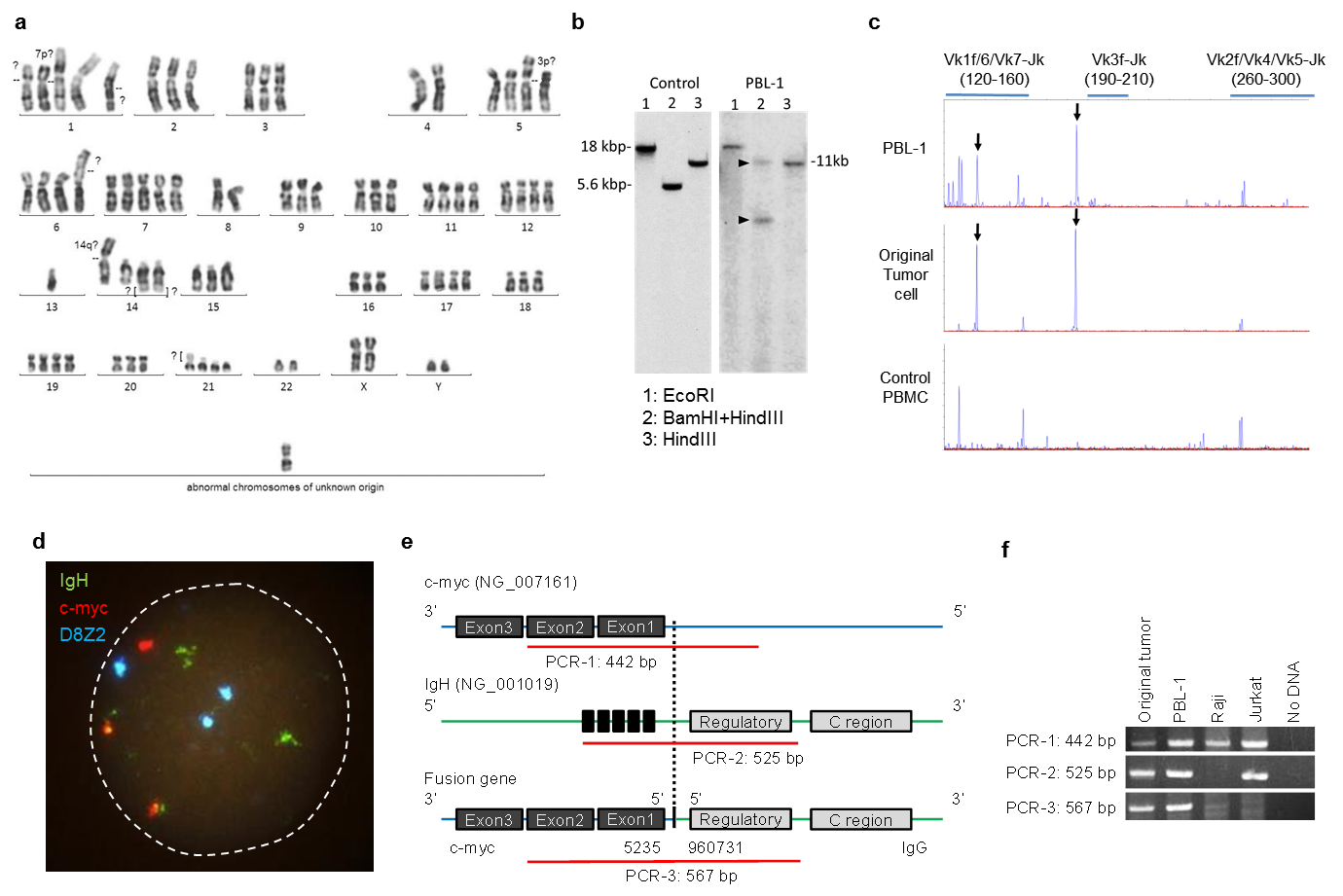


**Supplementary Figure 4.** Genomic character of PBL-1. (a) Chromosomal karyotype of PBL-1 cells. (b) Southern blot analysis of the IgH rearrangement. Arrowheads indicate monoclonal rearranged bands with a JH consensus probe. (c) Gene rearrangement of immunoglobulin κ chain by the BioMed2 method. Fragment analysis of PCR products by the BioMed 2 method is shown. Arrows indicate clonal bands. (d) Fluorescence *in situ* hybridization for the IgH/c-myc gene rearrangement. Two fusion (yellow), one c-myc (red), two IgH (green), and three D8Z2 (blue) signals were observed in 96% of cells examined. Outline of nucleus is indicated by a white broken line. (e) Gene maps of the IgH/c-myc fusion genome. C-myc (upper panel), IgG genome (middle panel), and fusion genome of IgH-c-myc (lower panel) are shown. A vertical break line indicates the break point. PCR-1-3 (red lines) indicate predicted PCR products in (f). (f) PCR products for breaking point of c-myc. PCR products of PCR-1-3 in (e) are electrophoresed in 2% ME agarose gel.

**Supplementary Figure 5.** Cell proliferation assay and flow cytometry for PBL-1. (a) BrdU assay in the IL-6 starvation. Absorbance of BrdU after IL-6 withdrawal are shown. Error bars indicate standard deviation. (b) BrdU assay in the addition of tocilizumab. Absorbance of BrdU and concentration of tocilizumab are indicated in the vertical and horizontal axes. Error bars indicate standard deviation. (c) Flow cytometry of Annexin V/ PI in addition of tocilizumab. Tocilizumab was added to the medium at 0.1 mg/mL. Hours after tocilizumab addition are shown at the top of each panel.


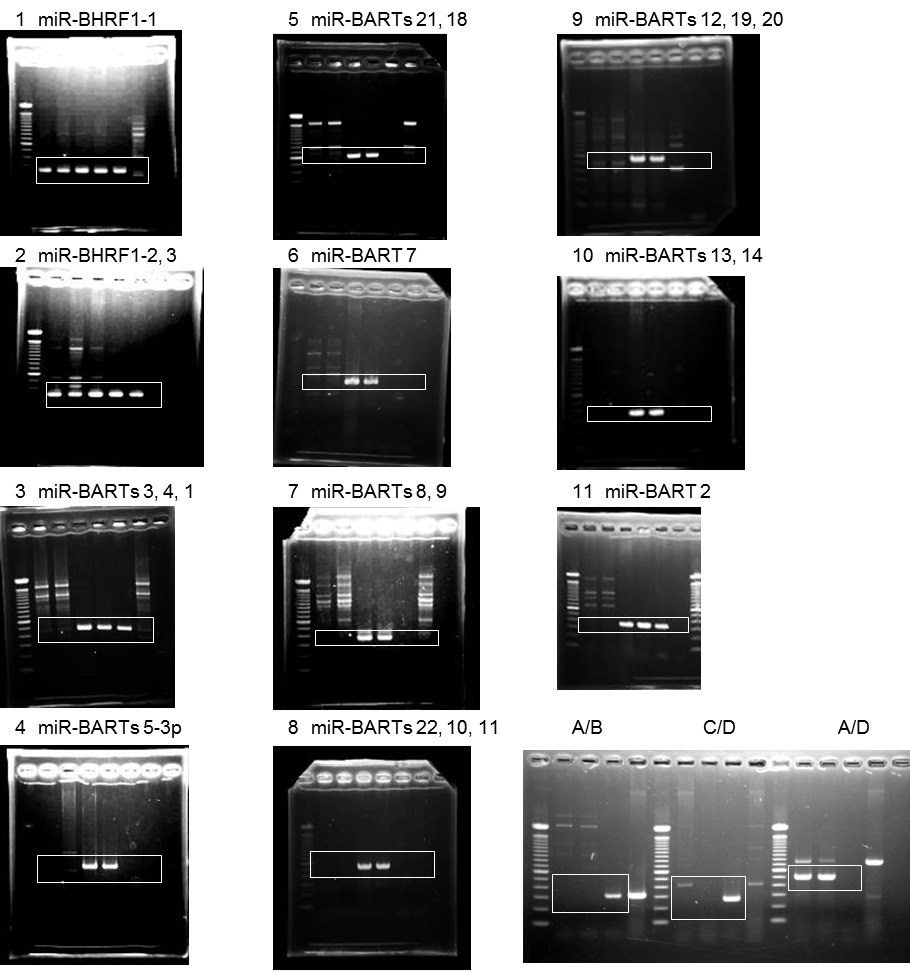


**Supplementary Figure 6.** Full length images of the cropped gels presented in main Figure 2.


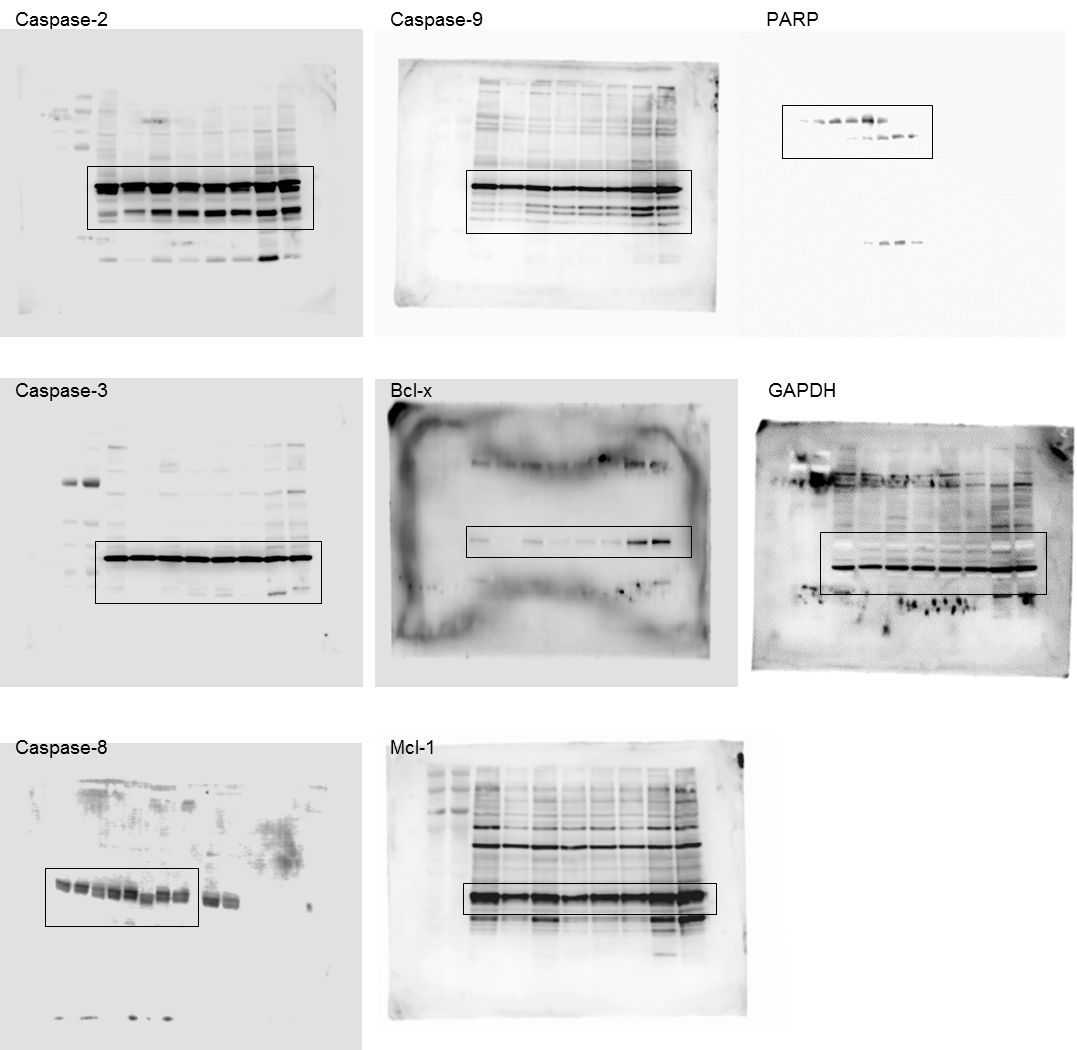


**Supplementary Figure 7.** Full length images of the cropped gels presented in main Figure 4c.

**
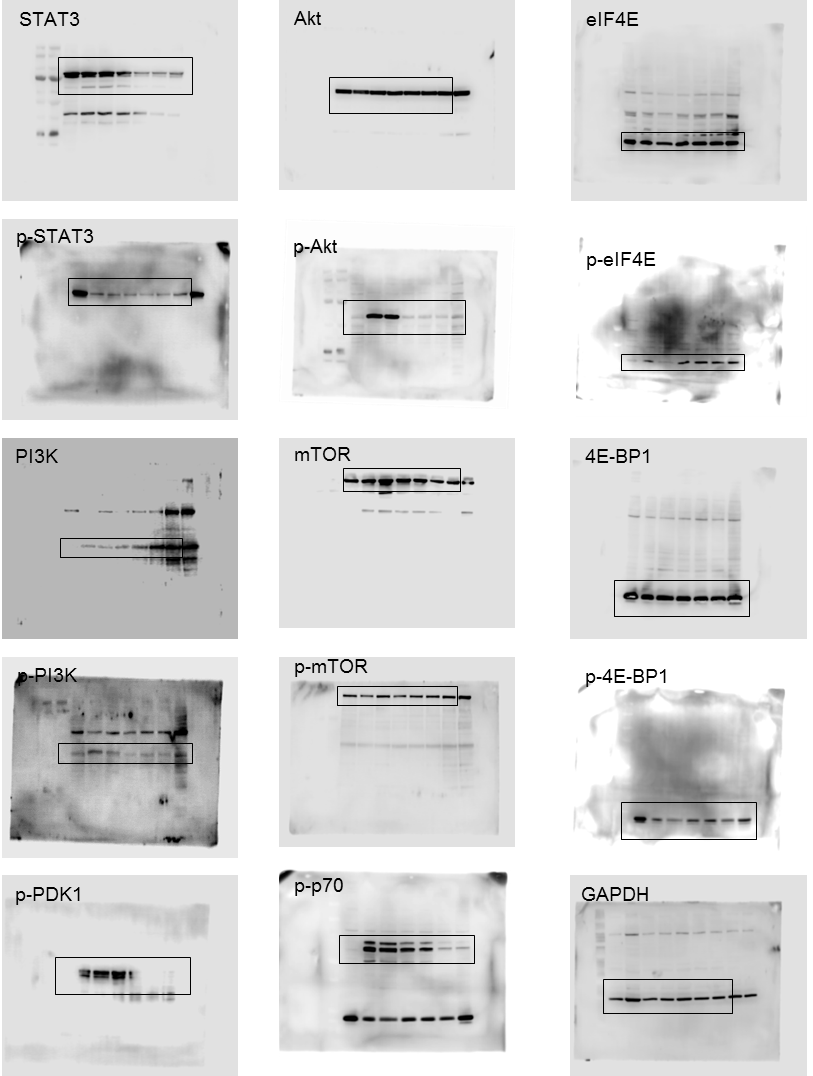
**

**Supplementary Figure 8.** Full length images of the cropped gels presented in main Figure 5a.
